# Update of Guidelines for laparoscopic treatment of ventral and incisional abdominal wall hernias (International Endohernia Society (IEHS)): Part B

**DOI:** 10.1007/s00464-019-06908-6

**Published:** 2019-07-10

**Authors:** R. Bittner, K. Bain, V. K. Bansal, F. Berrevoet, J. Bingener-Casey, D. Chen, J. Chen, P. Chowbey, U. A. Dietz, A. de Beaux, G. Ferzli, R. Fortelny, H. Hoffmann, M. Iskander, Z. Ji, L. N. Jorgensen, R. Khullar, P. Kirchhoff, F. Köckerling, J. Kukleta, K. LeBlanc, J. Li, D. Lomanto, F. Mayer, V. Meytes, M. Misra, S. Morales-Conde, H. Niebuhr, D. Radvinsky, B. Ramshaw, D. Ranev, W. Reinpold, A. Sharma, R. Schrittwieser, B. Stechemesser, B. Sutedja, J. Tang, J. Warren, D. Weyhe, A. Wiegering, G. Woeste, Q. Yao

**Affiliations:** 1 I.M. Sechenov First Moscow State Medical University of the Ministry of Health of the Russian Federation (Sechenov University), Trubetskaya str., 8, b. 2, Moscow, Russia 119992; 2 Emeritus Director, Marienhospital Stuttgart, Supperstr. 19, 70565 Stuttgart, Germany; 3 Department of Surgery, New York University, New York, USA; 4 Department of Surgical Disciplines, All India Institute of Medical Sciences, Room No. 5026A, 5th Floor, Teaching Block, Ansari Nagar, New Delhi, 110029 India; 5 Universitair Ziekenhuis Gent, C. Heymanslaan 10, 9000 Ghent, Belgium; 6 Division of Breast, Endocrine, Metabolic & Gastrointestinal Surgery, Mayo Clinic, 200 First Street SW, Rochester, MN 55905 USA; 7 Lichtenstein Amid Hernia Clinic at UCLA, Section of Minimally Invasive Surgery, UCLA Division of General Surgery, Los Angeles, USA; 8 Department of Hernia and Abdominal Surgery, Beijing Chao-Yang Hospital, Capital Medical University, Fengtai, China; 9 Max Super Speciality Hospital, 2 Press Enclave Road, Saket, New Delhi, 110017 India; 10 Klinik für Viszeral-, Gefäss- und Thoraxchirurgie, Kantonsspital Olten, Baslerstrasse 150, 4600 Olten, Switzerland; 11 Royal Infirmary of Edinburgh, Edinburgh, EH16 4SA UK; 12 Allgemein-, Viszeral- und Tumorchirurgie, Wilhelminenspital, 1160 Vienna, Austria; 13 ZweiChirurgen GmbH, Zentrum für Hernienchirurgie und Proktologie, St. Johanns-Vorstadt 44, 4056 Basel, Switzerland; 14 Department of Surgery, Mount Sinai Hospital, 1010 5th Avenue, New York, NY 10028 USA; 15 Department of Surgery, Southeast University School of Medicine, Main Add. 87 Ding Jia Qiao, Nanjing, 210009 Jiangsu China; 16 Digestive Disease Center, Bispebjerg Hospital, University of Copenhagen, 2400 Copenhagen NV, Denmark; 17 Visceral- und Gefäßchirurgie, Zentrum für Minimal Invasive Chirurgie, Vivantes Klinikum Spandau, Neue Bergstraße 6, 13585 Berlin, Germany; 18 Klinik Im Park, Grossmuensterplatz 9, 8001 Zurich, Switzerland; 19 Our Lady of the Lake Physician Group, 7777 Hennessy Blvd., Suite 612, Baton Rouge, LA 70808 USA; 20 Department of General Surgery, Ruijin Hospital, Shanghai Jiao Tong University School of Medicine, Shanghai, 200025 China; 21 Department of Surgery, YLL School of Medicine, National University Hospital, Level 2, Kent Ridge Wing 2, 5 Lower Kent Ridge Road, Singapore, 119074 Singapore; 22 Paracelsus Medizinische Universität Salzburg (PMU), Universitätsklinik für Chirurgie, Salzburg, Austria; 23 Mahatma Gandhi University of Medical Sciences & Technology, RIICO Institutional Area, Tonk Road, Sitapura, Jaipur, Rajasthan 302 022 India; 24 Centro de Cirugía Mayor Ambulatoria Ave María, Avda. de la Palmera, 53, 41013 Seville, Spain; 25 HANSECHIRURGIE, Niebuhr Marleschki & Partner, Alte Holstenstr. 16, 21031 Hamburg, Germany; 26 SUNY Downstate Medical Center, 450 Clarkson Avenue, Brooklyn, NY 11203 USA; 27 Department of Surgery, University Surgeons Associates, 1930 Alcoa Highway, Bldg A, Ste 285, Knoxville, TN 37920 USA; 28 Lenox Hill Hospital-Northwell Health, New York, USA; 29 Abteilung für Chirurgie, Wilhelmsburger Krankenhaus, Groß-Sand 3, 21107 Hamburg, Germany; 30 Abteilung für Chirurgie, LKH Hochsteiermark, Standort Bruck an der Mur Tragösser Str. 1, 8600 Bruck an der Mur, Austria; 31 Hernienzentrum Köln, Zeppelinstraße 1, 50667 Cologne, Germany; 32 Gading Pluit Hospital, Jl. Boulevard Timur Raya Kelapa Gading, Jakarta, 14250 Indonesia; 33 Department of General Surgery, Huadong Hospital, Fudan University, Shanghai, China; 34 Minimally Invasive Surgery, Greenville Health System, Department of Surgery, University of South Carolina School of Medicine, Greenville, USA; 35 Pius-Hospital Oldenburg, Klinik für Allgemein- und Viszeralchirurgie, Universitätsklinik für Viszeralchirurgie, Georgstraße 12, 26121 Oldenburg, Germany; 36 Department of General, Visceral, Vascular and Paediatric Surgery, University Hospital of Wuerzburg, Oberduerrbacher Strasse 6, 97080 Würzburg, Germany; 37 AGAPLESION ELISABETHENSTIFT gemeinnützige GmbH, Akademisches Lehrkrankenhaus, Landgraf-Georg-Strasse 100, 64287 Darmstadt, Germany; 38 Department of Hernia and Abdominal Surgery, Huashan Hospital, Fudan University, Shanghai, China

**Keywords:** Update Guidelines, Abdominal wall hernia, Ventral hernia repair, Primary ventral hernias, Secondary ventral hernias, Open sublay repair, Endoscopic sublay, Laparoscopic repair, IPOM, Rectus diastasis, Milos, Emilos, eTEP

## Abstract

**Abstract:**

In 2014 the International Endohernia Society (IEHS) published the first international “Guidelines for laparoscopic treatment of ventral and incisional abdominal wall hernias”. Guidelines reflect the currently best available evidence in diagnostics and therapy and give recommendations to help surgeons to standardize their techniques and to improve their results. However, science is a dynamic field which is continuously developing. Therefore, guidelines require regular updates to keep pace with the evolving literature.

**Methods:**

For the development of the original guidelines all relevant literature published up to year 2012 was analyzed using the ranking of the Oxford Centre for Evidence-Based-Medicine. For the present update all of the previous authors were asked to evaluate the literature published during the recent years from 2012 to 2017 and revise their statements and recommendations given in the initial guidelines accordingly. In two Consensus Conferences (October 2017 Beijing, March 2018 Cologne) the updates were presented, discussed, and confirmed. To avoid redundancy, only new statements or recommendations are included in this paper. Therefore, for full understanding both of the guidelines, the original and the current, must be read. In addition, the new developments in repair of abdominal wall hernias like surgical techniques within the abdominal wall, release operations (transversus muscle release, component separation), Botox application, and robot-assisted repair methods were included.

**Results:**

Due to an increase of the number of patients and further development of surgical techniques, repair of primary and secondary abdominal wall hernias attracts increasing interests of many surgeons. Whereas up to three decades ago hernia-related publications did not exceed 20 per year, currently this number is about 10-fold higher. Recent years are characterized by the advent of new techniques—minimal invasive techniques using robotics and laparoscopy, totally extraperitoneal repairs, novel myofascial release techniques for optimal closure of large defects, and Botox for relaxing the abdominal wall. Furthermore, a concomitant rectus diastasis was recognized as a significant risk factor for recurrence. Despite still insufficient evidence with respect to these new techniques it seemed to us necessary to include them in the update to stimulate surgeons to do research in these fields.

**Conclusion:**

Guidelines are recommendations based on best available evidence intended to help the surgeon to improve the quality of his daily work. However, science is a continuously evolving process, and as such guidelines should be updated about every 3 years. For a comprehensive reference, however, it is suggested to read both the initially guidelines published in 2014 together with the update. Moreover, the presented update includes also techniques which were not known 3 years before.

## Content Part B

Chapter 1: How can the new techniques for minimal invasive extraperitoneal mesh repair of abdominal wall hernias and rectus diastasis be defined?
Chapter 2: Is there an indication for operative treatment of diastasis recti without hernia formation?
Chapter 3: Component separation techniques.
Chapter 4: In which patient group is a transversus abdominis release (TAR) indicated?
Chapter 5: The role of preoperative adjunct interventions in ventral hernia repair
Chapter 6: Robotic ventral/incisional hernia repair
Chapter 7: Treatment of lateral primary or incisional hernias: Which technique should be preferred?
Chapter 8: Education and Training in Laparoscopic Ventral Hernia Repair.

## Introduction

Treatment of abdominal wall hernias is a rapidly evolving field of surgery. Correspondingly there is a dramatic increase of publications. There are many reasons for this development: rise of the number of laparotomies and the number of major surgeries being performed, progress in anesthesiology, increase of older patients with weak connective tissue, increase of patients with risk factors for hernias, and significant increase of patients managed with an open abdomen in a damage-control situation. Worldwide as many as two million patients are operated on every year. A variety of new repair techniques came up, recently even robot-assisted operations. The surgical approach may be open, laparoscopic, endoscopically within the abdominal wall, or hybrid approaches combining these modalities. The volume of literature, often with low levels of evidence and conflicting results, can be difficult to interpret in a meaningful way to assist the surgeon in appropriate management of the hernia patient. Therefore, there is a need for evidence-based guidelines to help the surgeon in his daily decision making process. “Guidelines are the bridge between science and clinical practice (Eccles M, Mason J.Health Technol Assess. 2001; 5(16):1–69. Review.). In 2014 this same group (IEHS) published the first international “Guidelines for laparoscopic treatment of ventral and incisional abdominal wall hernias” [1, 2, 3]. It is generally accepted that guidelines require an update every 3 years to reflect the rapid evolution of techniques, materials and data available. The current update follows the same methodology as described in the original guidelines. The authors were encouraged to avoid redundancy and concentrate on the new studies showing a level of evidence 1, 2 and 3, and which were published between 2012 and 2017. Statements and recommendations which are still valid are not repeated. As such, this update should be read in the context and in conjunction with the initially published guidelines. New topics included in this update are: In which patient group is a component separation indicated? Should the component separation be done open or endoscopically? Is an anterior component separation better than the posterior one? Is preliminary treatment with Botox indicated in patients in whom a component separation is planned? Should TAR be done open or endoscopically? In patients presenting with a ventral hernia in combination with a rectus diastasis which is the best treatment option? Does robot- assisted surgery have a future in repair of primary and secondary ventral hernias? What is the optimal treatment of lateral primary or incisional hernias? We are well aware that with respect to these innovations the evidence is not yet strong enough to give valuable statements or recommendations, however, the guidelines should inform the surgical community and stimulate further studies to gain more knowledge in the coming years.

**References**

1. Bittner R, Bingener-Casey J, Dietz U, Fabian M, Ferzli GS, Fortelny RH, Köckerling F, Kukleta J, Leblanc K, Lomanto D, Misra MC, Bansal VK, Morales-Conde S, Ramshaw B, Reinpold W, Rim S, Rohr M, Schrittwieser R, Simon T, Smietanski M, Stechemesser B, Timoney M, Chowbey P; International Endohernia Society (IEHS) (2014) Guidelines for laparoscopic treatment of ventral and incisional abdominal wall hernias (International Endohernia Society (IEHS)-part 1. Surg Endosc Jan; 28(1):2–29. 10.1007/s00464-013-3170-6. Epub 2013 Oct 11
2. Bittner R, Bingener-Casey J, Dietz U, Fabian M, Ferzli GS, Fortelny RH, Köckerling F, Kukleta J, Leblanc K, Lomanto D, Misra MC, Bansal VK, Morales-Conde S, Ramshaw B, Reinpold W, Rim S, Rohr M, Schrittwieser R, Simon T, Smietanski M, Stechemesser B, Timoney M, Chowbey P; International Endohernia Society (IEHS) (2014) Guidelines for laparoscopic treatment of ventral and incisional abdominal wall hernias (International Endohernia Society (IEHS)-part 2. Surg Endosc Feb;28(2):353–79.
3. Bittner R, Bingener-Casey J, Dietz U, Fabian M, Ferzli GS, Fortelny RH, Köckerling F, Kukleta J, Leblanc K, Lomanto D, Misra MC, Bansal VK, Morales-Conde S, Ramshaw B, Reinpold W, Rim S, Rohr M, Schrittwieser R, Simon T, Smietanski M, Stechemesser B, Timoney M, Chowbey P; International Endohernia Society (IEHS) (2014) Guidelines for laparoscopic treatment of ventral and incisional abdominal wall hernias (International Endohernia Society (IEHS)-part 3. Surg Endosc Feb;28(2):380–404. 10.1007/s00464-013-3172-4. Epub 2013 Sep 17

## Chapter 1. Key question: how can the new techniques for minimal invasive extraperitoneal mesh repair of abdominal wall hernias and rectus diastasis be defined?

### David Chen, Wolfgang Reinpold, Reinhard Bittner, Ferdinand Koeckerling

**Methodology**

Search of MEDLINE, Embase, PubMed, PubMed Central, The Cochrane central registry of controlled trials (CENTRAL), Google Scholar, and Springer Link

**Search terms**

“incisional hernia”, “abdominal wall hernia”, “laparoscopic sublay repair”, endoscopic sublay mesh repair”, “endoscopic preperitoneal mesh repair”, “laparoscopic preperitoneal mesh repair”, “laparoscopic extraperitoneal mesh repair”, “sublay mesh repair”, “preperitoneal mesh repair”, “retromuscular mesh repair”, “endoscopic retromuscular mesh repair”, “laparoscopic retromuscular “ventral hernia”, “laparoscopic ventral hernia”, “endoscopic ventral hernia”, “laparoscopic umbilical hernia”, “ELAR”, “MILOS”, “EMILOS”, “eTEP”, “rTAPP ventral hernia”, “rRives hernia”, “TAR”, “rTAR”, “Stapler Abdominoplasty”, “stapled closure”, “Robotic”, “midline augmentation”, “midline reconstruction”, “Rectus diastasis”, “minimal invasive ventral hernia”, “minimal invasive incisional hernia”, “minimal invasive abdominal wall hernia”.

**Included publications**

Covering the period from 2003 to February 2018, using the search terms “abdominal wall hernia”, “ventral hernia”, and “incisional hernia” identified 12,507, 9971, and 3806 articles, respectively. One hundred and forty-three articles were identified for “rectus diastasis”. These were refined to 89 studies. In total, 30 studies were found to be relevant. Twenty-three were included in formulating these guidelines while 7 were excluded for language (2), open technique (2), or low quality (3).

Table 1 Classification of new techniques for minimal invasive extraperitoneal mesh repair of abdominal wall ventral hernias
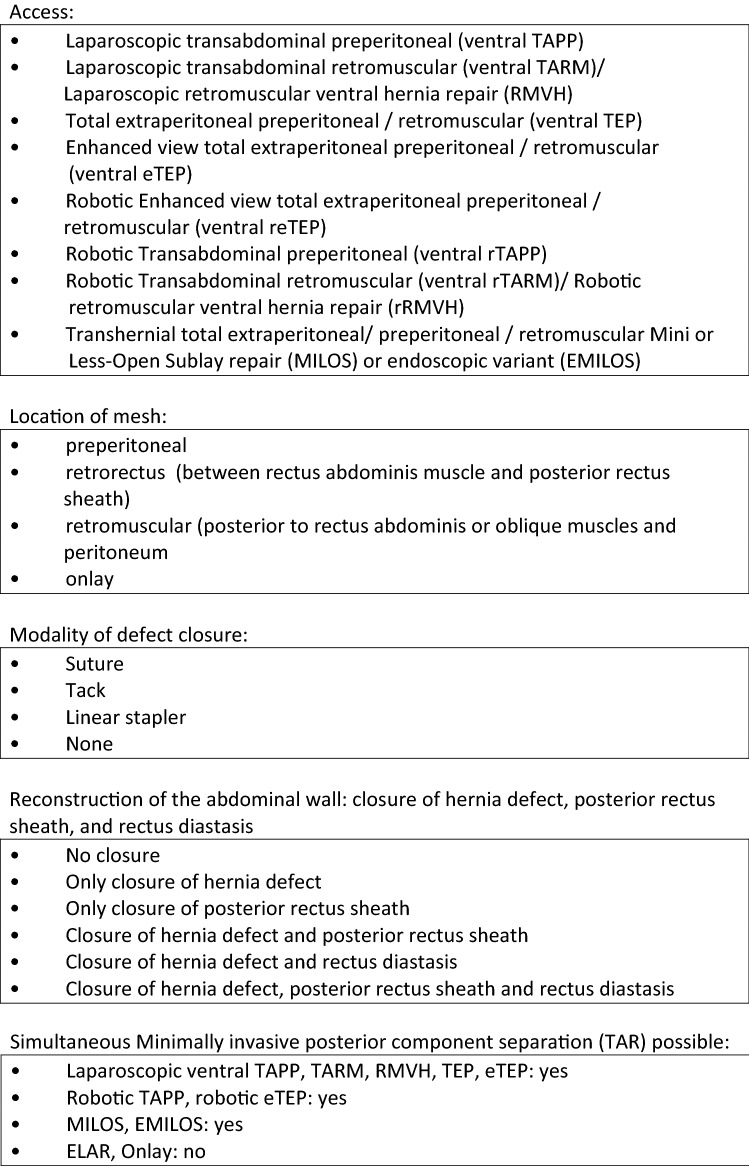


**Evidence of new minimal invasive extraperitoneal mesh repair techniques**


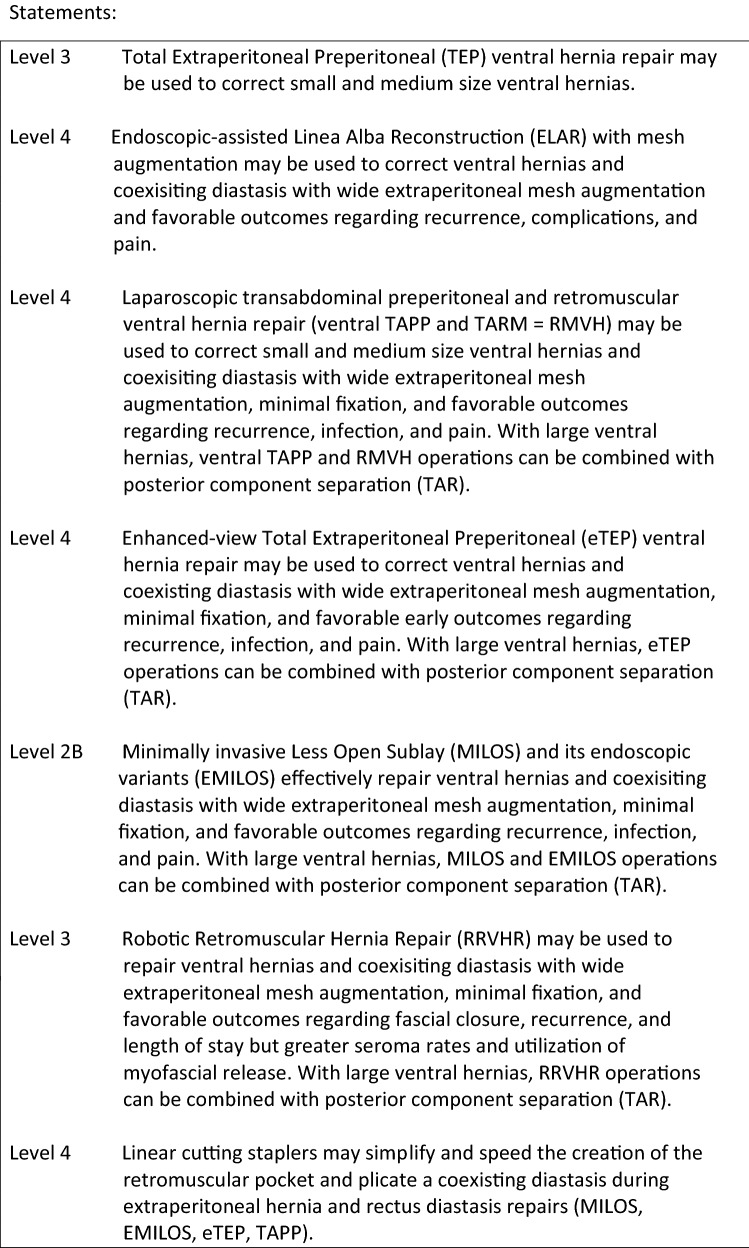



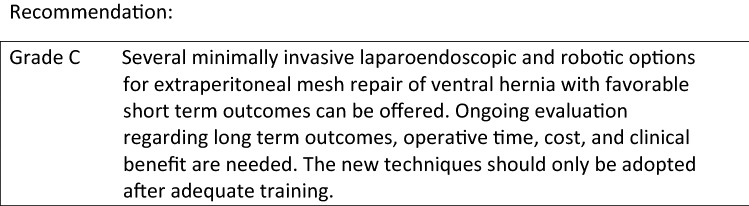


**Key question**

In patients presenting with a ventral hernia in combination with a rectus diastasis, which is the best treatment option- IPOM plus, ELAR, MILOS, EMILOS, LIRA, eTEP, Stapler Abdominoplasty?


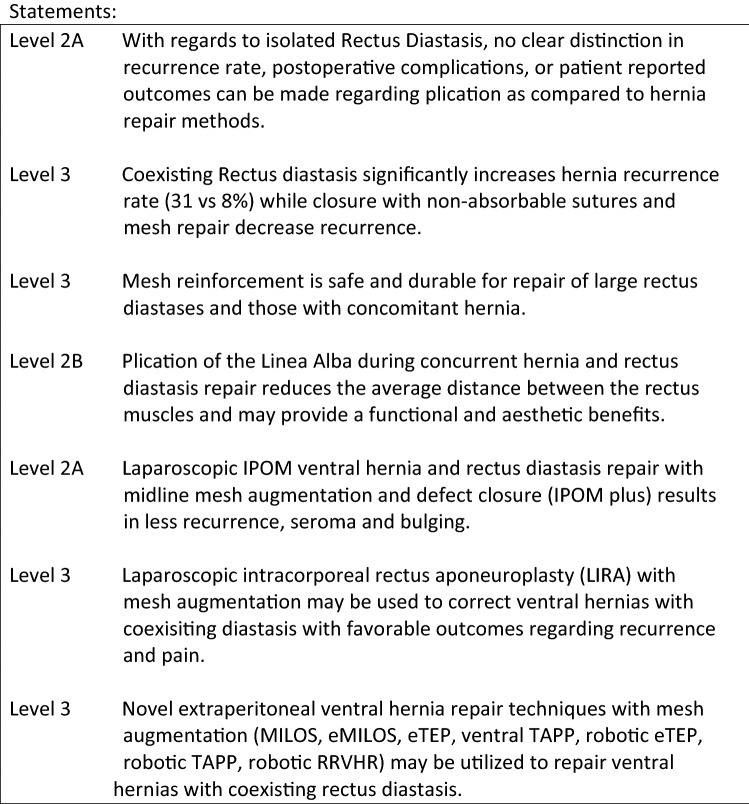



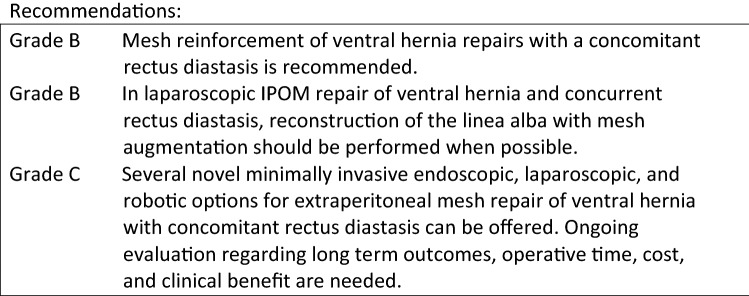


**Introduction**

Laparoscopic intraperitoneal onlay mesh (IPOM) repair and open sublay mesh repair are currently the most common techniques for the treatment of primary and recurrent abdominal wall hernias worldwide [1–3]. A systematic review and two recently published meta-analyses concluded that laparoscopic IPOM and open abdominal wall hernia repairs are safe procedures with comparable short and long-term outcomes [1–3]. Although the open techniques are burdened with higher infection rates, laparoscopic IPOM repairs carry an increased risk of intraoperative bowel injury, adhesions, and bowel obstruction (1–4). Despite progress in mesh technology and the development of coated meshes designed to lower the risk of adhesion formation, the potential risks associated with an intraperitoneal foreign body have not yet been eliminated [1–4]. Traumatic mesh fixation increases the risk of adhesions, visceral damage, nerve injury, and acute and chronic pain. Reduction of the hernia sac with closure of the hernia defect is difficult with laparoscopic IPOM, and is often omitted leading to higher recurrence rates, eventrations (pseudorecurrences), and seroma formation [5–8]. In larger hernias with a diameter of more than 15 cm, the laparoscopic IPOM repair can be very difficult [1–7].

To address the limitations of traditional laparoscopic and open ventral hernia repair, several minimally invasive endoscopic, laparoscopic, and robotic extraperitoneal techniques have developed with the goal of combining the benefits of traditional open sublay repair removing prosthetics out of the visceral compartment with those of minimally invasive surgery. The new minimal invasive extraperitoneal techniques can be classified according to access, mesh location, modality of defect closure, and anatomic reconstruction of the abdominal wall. In all of these novel procedures, standard uncoated permanent synthetic meshes (Polypropylene, PVDF, Polyester) may be used. Analogous to the differentiation in laparoscopic inguinal hernia repair, laparoscopic transabdominal techniques (ventral preperitoneal TAPP, ventral transabdominal retromuscular TARM/RMVH) can be differentiated from endoscopic total extraperitoneal procedures (ventral TEP, eTEP, MILOS, EMILOS). Most of these novel extraperitoneal operations can be combined with posterior component separation (transversus abdominis release—TAR) as needed to address larger defects or accommodate larger prostheses. The first article on minimal invasive extraperitoneal mesh repair of ventral hernias was published by Miserez et al. [8]. Subsequent development of transabdominal, total extraperitoneal, transhernial, and onlay techniques perfomed in mini-open, laparoscopic, and robotic fashion have expanded the minimally invasive extraperitoneal options for repair of ventral hernias and coexisting rectus diastasis. Classification details for these novel extraperitoneal repairs are shown in Table 1.

Patients with symptomatic umbilical, ventral, and incisional hernias and concomitant rectus abdominis diastasis represent a growing clinical problem and, as with isolated hernias, the ideal operative management of this complex hernia situation has not been defined. With regards to isolated rectus diastasis, defined as a thinning and widening of the linea alba, combined with laxity of the ventral abdominal wall musculature, controversy remains as to whether this pathology is cosmetic or functional due to the variation in severity, symptomatology, and the absence of strangulation risk found with hernias [9]. While physiotherapy can achieve a limited benefit with regards to size and symptoms with isolated mild rectus diastasis, surgery significantly improves abdominal wall function and pain regardless of operative technique employed and should be considered with diastasis wider than 3 cm [10]. Currently, both plication and hernia repair methods are used to repair isolated rectus abdominis diastasis with no clear distinction in recurrence rate, postoperative complications, or patient reported outcomes found in the literature [9, 10].

The presence of rectus abdominis diastasis and coexisting hernia presents a greater challenge as the weakened linea alba and ventral abdominal musculature increase the risk of hernia formation, compromise the integrity of the midline, and complicate the repair of ventral hernias. Köhler et al. demonstrated that a coexisting rectus diastasis significantly increases hernia recurrence rate with primary sutured closure (31 vs. 8%) [11]. As such, well established principles from ventral hernia repair including primary closure with non-absorbable sutures and mesh repair should be utilized to decrease hernia and diastasis recurrence [11]. Mesh repair of ventral hernias is well established as safe reducing the recurrence rate significantly compared to suture repair. Based on the volume of Level 1 studies studying ventral hernia, mesh reinforcement of ventral hernia repairs with a concomitant rectus diastasis is recommended while studies specifically evaluating ventral hernia and rectus diastasis corroborate these findings [12].

While the role of defect closure with isolated ventral hernia is likely beneficial but still unestablished, mesh reinforcement with ventral hernia in the setting of a concurrent rectus diastasis is more clear [11]. Multiple studies utilizing several different operative repair techniques demonstrate that restoration of the linea alba during concurrent hernia and rectus diastasis repair is feasible and reduces the average distance between the rectus muscles with potential functional and esthetic benefits [13–26]. These studies include traditional laparoscopic intraperitoneal (IPOM) repair with defect closure, subcutaneous closure of the anterior sheath, endoscopic subaponeurotic closure of the anterior sheath, laparoscopic sutured linea alba closure, laparoscopic posterior sheath closure, and linear stapled closure techniques to address the separation of the linea alba prior to mesh hernia repair. Restoration of the midline with correction of the diastasis at the time of ventral hernia repair when possible may provide additional benefit with regards to abdominal wall function, pain, and cosmesis [5, 10, 13, 17, 22, 25].

With regard to specific technique, several of the new minimally invasive mini-open, endoscopic, laparoscopic, and robotic extraperitoneal ventral hernia repairs may simultaneously address a coexisting rectus diastasis. There is a paucity of data in the literature for many of these novel techniques and an absence of comparative data to establish superiority of any given technique. The current literature summarized in these guidelines support that these reported techniques are safe, feasible, and effective in shorter term studies but ongoing and future studies are need to establish a “best treatment option”.

Laparoscopic intraperitoneal onlay mesh (IPOM) repair is the best and longest studied minimally invasive ventral hernia repair technique. Palanivelu et al. described their “Venetian blind” technique in 2009 to address the cosmetic and functional implications of a coexisting rectus diastasis or bridged hernia defect demonstrating the feasibility and efficacy of closing the defect at the time of IPOM repair [13]. Multiple primary studies, meta-analyses, and systematic reviews have subsequently been performed demonstrating the benefits of defect closure with midline mesh augmentation during IPOM repair with regards to recurrence, seroma, and “pseudorecurrence” or bulging [5–7]. Extrapolating the data from these consistent level 2A studies to concurrent rectus diastasis and ventral hernia repair, reconstruction of linea alba with mesh augmentation, is recommended and should be performed when feasible.

The minimally invasive Mini or Less Open Sublay Repair (MILOS) and its endoscopic variant (EMILOS) developed by Reinpold and colleagues utilize progressively smaller “mini (≤ 5 cm) or less open (6–12 cm)” incisions and laparoendoscopic transhernial approaches to replicate the traditional open Rives-Stoppa retrorectus or retromuscular sublay reconstruction for ventral hernia repair [14–16]. This approach simultaneously addresses the midline linea alba correcting coexisting diastasis with wide midline mesh augmentation and minimal fixation. Reinpold et al. performed a prospective, propensity score matched study within the German Hernia Registry (Herniamed) comparing 615 MILOS incisional hernia operations to laparoscopic IPOM incisional hernia repair and open sublay repair [14]. MILOS repair was associated with significantly fewer postoperative surgical complications, general complications, recurrences, and less chronic pain versus IPOM repair. Significantly fewer postoperative complications, reoperations, infections, general complications, recurrences, and less chronic pain were found compared to open sublay repair. The MILOS technique reproduces the functional and physiologic aspect of an open retromuscular repair with the benefits of minimally invasive techniques. Schwarz et al. and Bittner et al. demonstrated that this technique can be endoscopically modified (EMILOS) with similar feasibility, efficacy, and favorable outcomes for ventral hernia and rectus diastasis repair [15, 16]. In large defects, the MILOS technique can be combined with a posterior component separation (TAR).

The Enhanced or Extended-view Total Extraperitoneal Preperitoneal (eTEP) approach developed by Daes has been used to perform midline and off midline ventral hernia repair augmenting the traditional preperitoneal space utilized in inguinal hernia repair to access almost any portion of the abdominal wall. With regards to ventral hernia with a coexisting rectus diastasis, this technique allows for midline closure of the linea alba and any diastasis with wide extraperitoneal mesh augmentation, minimal fixation, and may be combined with posterior component separation with release of the transversus abdominus muscle to access to the entire retromuscular plane. Belayansky et al. performed a retrospective multicenter review of 79 patients utilizing the eTEP approach demonstrating low complication rates, significantly improved pain and functionality scores using the Carolinas Comfort Scale, and low complication, infection, and recurrence rates [17]. While the operative approach may differ slightly, the eTEP technique is anatomically and physiologically similar to the eMILOS technique demonstrating similar benefits and efficacy.

The Endoscopic-assisted Linea Alba Reconstruction (ELAR) with mesh augmentation developed by Koeckerling is based on the principles of the MILOS technique and utilizes an endoscopic subcutaneous approach with subsequent mesh augmentation and reinforcement to address ventral hernia defects with concurrent rectus diastasis [20]. This hybrid technique exposes the anterior layer of the rectus sheath from the xiphoid process to the subumbilical area, the medial segments of the anterior layer of both rectus sheaths are sutured to reconstruct the linea alba, and the resultant defect in the anterior layer of the rectus sheath is repaired by suturing a mesh to the anterior sheaths bilaterally to reconstruct the anterior abdominal wall. Koeckerling et al. reported low complication and reoperation rates (1.4%, 2 cases due to bleeding) with favorable early results regarding pain and recurrence in their series of 140 patients [18–20].

Laparoscopic intracorporeal rectus aponeuroplasty (LIRA) with mesh augmentation described by Gómez-Menchero et al. was designed to address some of the challenges and limitations of closing the defect with the IPOM plus technique which may increase pain, recurrence, or require component separation to counteract the tension created by midline fascial reapproximation [21]. The posterior rectus aponeurosis is laparoscopically opened lengthwise around the hernia defect to create two flaps. The flaps are then sutured closed and the repair reinforced with an IPOM mesh. In their series of 12 patients followed to 1 year, this novel technique achieves a reproducible, feasible, “tension-free” repair of ventral hernias with coexisting rectus diastasis with a low rate of postoperative pain and no reported recurrence, or bulging [21].

Despite its benefits, minimally invasive laparoscopic ventral hernia repair can be technically challenging especially with complex procedures such as component separation, transversus abdominis release, and suturing of the ventral midline. The need for high technical skill, dexterity, and proficiency may lead to variability with regards to clinical outcomes, complication rates, and adoption. The use of the robotic platform for ventral hernia has progressively increased due to the benefits of improved optics and visualization, superior ergonomics, and improved degrees of freedom and range of motion needed to perform precise suturing, adhesiolysis, dissection, and mesh positioning and fixation necessary for abdominal wall reconstruction. With regards to ventral hernias and coexisting rectus diastasis, the Robotic Retromuscular Hernia Repair (RRVHR) utilizes the same operative plane as the open Rives-Stoppa, MILOS, eMILOS, and eTEP ventral hernia repairs. Warren et al. compared their robotic retromuscular repair (53 patients) to traditional laparoscopic ventral hernia repair (103 patients) utilizing registry data from the American Hernias Society Quality Collaborative. Robotic retromuscular repair facilitated facial closure and extraperitoneal mesh placement in the vast majority of cases (96.2 vs. 50.5%; *p* < 0.001). Hernia size was similar but robotic operations were longer and myofascial release was performed in 43% of robotic operations. Direct hospital costs were similar between both groups while length of stay was significantly shorter after RRVHR (23). Robotic retromuscular ventral hernia repair enables a true abdominal wall reconstruction and allows for simultaneous correction of coexisting rectus diastasis with wide extraperitoneal mesh augmentation, minimal fixation, and favorable outcomes regarding fascial closure and recurrence [23].

The term “Stapler abdominoplasty” represents a technical modification of several extraperitoneal operative techniques for ventral hernia and rectus diastasis repair rather than a unique operative approach. Linear cutting staplers may be used to develop the retromuscular space and plicate a coexisting diastasis during extraperitoneal hernia and rectus diastasis repairs. Costa et al. introduced this technique using a laparoscopic transabdominal preperitoneal approach in 15 postbariatic patients with hernia and rectus diastasis demonstrating feasibility, simplicity, and low complication rates [7]. Stapled plication of the anterior rectus sheath and division of the linea alba into an anterior and posterior component may be used in conjunction with open Rives-Stoppa, MILOS, EMILOS, eTEP, and laparoscopic retromuscular repairs [24–26].

In general, the indication for repair of diastasis recti of the abdominal muscles is based upon cosmetic or functional impairment, as there is no risk of strangulation. However, the negative implication of a rectus diastasis on ventral hernias with regards to recurrence and complications warrants consideration of the optimal approach to address both disease processes simultaneously. In these cases, the morbidity and risk of larger, more complicated operations for typically smaller coexisting hernias must be considered. Currently, there are several novel minimally invasive endoscopic, laparoscopic, and robotic procedures to address ventral hernia with coexisting rectus diastasis that are feasible, safe, and effective. Future studies with long-term outcomes and comparative data will help to define optimal techniques for these coexisting pathologies.

**References** (in parentheses the level of evidence)

1. Sauerland S, Walgenbach M, Habermalz B, Seiler CM, Miserez M (2011) Laparoscopic versus open surgical techniques for ventral or incisional hernia repair. Cochrane Database Syst Rev 16(3):CD007781. (2)
2. Al Chalabi H, Larkin J, Mehigan B, McCormick P (2015) A systematic review of laparoscopic versus open abdominal incisional hernia repair, with meta-analysis of randomized controlled trials. Int J Surg 20:65–74 (**2**)
3. Awaiz A, Rahman F, Hossain MB, Yunus RM, Khan S, Memon B, Memon MA (2015) Meta-analysis and systematic review of laparoscopic versus open mesh repair for elective incisional hernia. Hernia 19(3):449–463 (**2**)
4. Arita NA, Nguyen MT, Nguyen DH, Berger RL, Lew DF, Suliburk JT, Askenasy EP, Kao LS, Liang MK (2015) Laparoscopic repair reduces incidence of surgical site infections for all ventral hernias. Surg Endosc 29(7):1769–1780. (**3**)
5. Mitura K, Skolimowska-Rzewuska M, Garnysz K (2017) Outcomes of bridging versus mesh augmentation in laparoscopic repair of small and medium midline ventral hernias. Surg Endosc 31(1):382–388. Epub 2016 Jun 10. (**2B**)
6. Nguyen DH, Nguyen MT, Askenasy EP, et al. (2014) Primary fascial closure with laparoscopic ventral hernia repair: Systematic review. World J Surg 38:3097–3104 (**2A**)
7. Tandon A, Pathak S, Lyons NJ, Nunes QM, Daniels IR, Smart NJ (2016) Meta-analysis of closure of the fascial defect during laparoscopic incisional and ventral hernia repair. Br J Surg 103(12):1598–1607. Epub 2016 Aug 22. (**2A**)
8. Miserez M1, Penninckx F (2002) Endoscopic totally preperitoneal ventral hernia repair. Surg Endosc 16(8):1207–13. (**4**)
9. Mommers EHH, Ponten JEH, Al Omar AK, de Vries Reilingh TS, Bouvy ND, Nienhuijs SW (2017) The general surgeon’s perspective of rectus diastasis. A systematic review of treatment options. Surg Endosc 31(12):4934–4949. 10.1007/s00464-017-5607-9. Epub 2017 Jun 8 (**2A**)
10. Emanuelsson P, Gunnarsson U, Dahlstrand U, Strigård K, Stark B (2016) Operative correction of abdominal rectus diastasis (ARD) reduces pain and improves abdominal wall muscle strength: A randomized, prospective trial comparing retromuscular mesh repair to double-row, self-retaining sutures. Surgery 160(5):1367–1375. Epub 2016 Jul 27 PMID: 27475817 (**1B**)
11. Köhler G, Luketina RR, Emmanuel K (2015) Sutured repair of primary small umbilical and epigastric hernias: concomitant rectus diastasis is a significant risk factor for recurrence. World J Surg 39(1):121–126; discussion 127. PMID: 25217109 (**4**)
12. Cheesborough JE, Dumanian GA (2015) Simultaneous prosthetic mesh abdominal wall reconstruction with abdominoplasty for ventral hernia and severe rectus diastasis repairs. Plast Reconstr Surg 135(1):268–276. PMID: 25539311 (**4**)
13. Palanivelu C, Rangarajan M, Jategaonkar PA, Amar V, Gokul KS, Srikanth B (2009) Laparoscopic repair of diastasis recti using the ‘Venetian blinds’ technique of plication with prosthetic reinforcement: a retrospective study. Hernia 13(3):287–292. Epub 2009 Feb 12. PMID: 19214651 (**4**)
14. Reinpold W, Schröder M, Berger C, Nehls J, Schröder A, Hukauf M, Köckerling F, Bittner R (2018) Mini- or Less-open Sublay Operation (MILOS): A New Minimally Invasive Technique for the Extraperitoneal Mesh Repair of Incisional Hernias. Ann Surg Jan 16. 10.1097/sla.0000000000002661. [Epub ahead of print] (**2**)
15. Schwarz J, Reinpold W, Bittner R (2017) Endoscopic mini/less open sublay technique (EMILOS)-a new technique for ventral hernia repair. Langenbecks Arch Surg 402(1):173–180. (**3**)
16. Bittner R, J. Schwarz J (2017) Endoscopic mini/less open sublay operation for treatment of primary and secondary ventral hernias of the abdominal wall. Europ Surg (**3**)
17. Belyansky I, Daes J, Radu VG, Balasubramanian R, Reza Zahiri H, Weltz AS, Sibia US, Park A, Novitsky Y (2018) A novel approach using the enhanced-view totally extraperitoneal (eTEP) technique for laparoscopic retromuscular hernia repair. Surg Endosc 32(3):1525–1532. 10.1007/s00464-017-5840-2. Epub 2017 Sep 15 (**3**)
18. Köckerling F, Botsinis MD, Rohde C, Reinpold W, Schug-Pass C (2017) Endoscopic-assisted linea alba reconstruction: New technique for treatment of symptomatic umbilical, trocar, and/or epigastric hernias with concomitant rectus abdominis diastasis. Eur Surg 49(2):71–75. 2017 Mar 10. PMID: 28408920 (**3**)
19. Köckerling F, Botsinis MD, Rohde C, Reinpold W (2016) Endoscopic-Assisted Linea Alba Reconstruction plus Mesh Augmentation for Treatment of Umbilical and/or Epigastric Hernias and Rectus Abdominis Diastasis—Early Results. Front Surg 13;3:27. (**3**)
20. Köckerling F, Botsinis MD, Rohde C, Reinpold W, Schug-Pass C (2017) Endoscopic-assisted linea alba reconstruction. Eur Surg 49(2):71–75. 10.1007/s10353-017-0473-1. Epub 2017 Mar 10 (**3**)
21. Gómez-Menchero J, Guadalajara Jurado JF, Suárez Grau JM, Bellido Luque JA, García Moreno JL, Alarcón Del Agua I, Morales-Conde S (2018) Laparoscopic intracorporeal rectus aponeuroplasty (LIRA technique): a step forward in minimally invasive abdominal wall reconstruction for ventral hernia repair (LVHR). Surg Endosc 32(8):3502–3508. 10.1007/s00464-018-6070-y. Epub 2018 Jan 17 (**3**)
22. Bellido Luque J, Bellido Luque A, Valdivia J, Suarez Gráu JM, Gomez Menchero J, García Moreno J, Guadalajara Jurado J (2015) Totally endoscopic surgery on diastasis recti associated with midline hernias. The advantages of a minimally invasive approach. Prospective cohort study. Hernia 19(3):493–501. Epub 2014 Aug 21. PMID: 25142493 (**3**)
23. Warren JA, Cobb WS, Ewing JA, Carbonell AM (2017) Standard laparoscopic versus robotic retromuscular ventral hernia repair. Surg Endosc 31(1):324–332. Epub 2016 Jun 10. PMID: 27287903 (**3**)
24. Costa TN, Abdalla RZ, Santo MA, Tavares RR, Abdalla BM, Cecconello I (2016) Transabdominal midline reconstruction by minimally invasive surgery: technique and results. Hernia 20(2):257–265. PMID: 26801185 (**4**)
25. Moore AM, Anderson LN, Chen DC (2016) Laparoscopic Stapled Sublay Repair With Self-Gripping Mesh: A Simplified Technique for Minimally Invasive Extraperitoneal Ventral Hernia Repair. Surg Technol Int. 2016 Oct 26;29:131–139. (**4**)
26. Nguyen DK, Chen DC (2017) Laparoendoscopic stapled Rives Stoppa sublay technique for extraperitoneal ventral hernia repair. Europ Surg 49:175–179. (**4**)

## Chapter 2. Is there an indication for operative treatment of diastasis recti without hernia formation?

### Mazen Iskandar MD, Dimitri Ranev, MD, and George Ferzli, MD

The following search terms were used: “diastasis recti” or “diastasis rectus” or “diastasis of the recti” or “diastasis of the rectus” or “rectus diastasis” or “rectus abdominis diastasis” or “divarication”. A systemic review of the literature was done in September 2017. A total of 219 papers were found. After selecting relevant studies, 20 articles were used for this review.


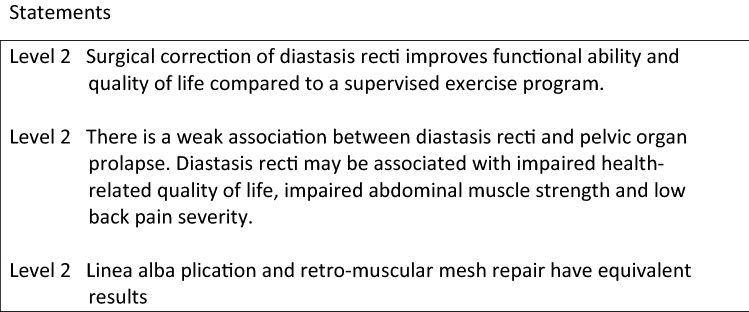



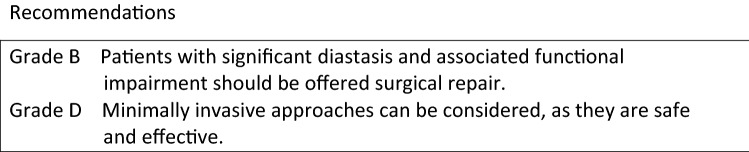


**Introduction**

Diastasis recti is a diagnosis that surgeons have to manage on a regular basis. It is simply described as bulging of the linea alba with increased intra–intra-abdominal pressure. Typically, women during pregnancy develop diastasis recti which regresses within a year after child birth. However, in a third of those patients, the diastasis persists [1]. The width of the linea alba is measured at 2 reference points. The first is at 3 cm superior to the umbil-icus and the second is 2 cm inferior. A patient is considered to have diastasis if the width between the 2 medial edges of the recti is greater than 22 mm at the first reference point and greater than 16 mm at the second point [2]. Nahas proposed a classification that included 4 types of “myofascial deformities” with a tailored treatment for each type [3]. In addition to diastasis recti, these types took into consideration other factors such as the appearance of the waist line and the presence of lateral laxity. Most women with diastasis recti after pregnancy fall under type A and are treated with plication of the anterior rectus sheath.

**Indications for surgery**

Diastasis recti, even when associated with significant protrusion, does not represent a true hernia and poses no risk of incarceration or strangulation. Thus, the decision for surgery is based on functional and/or cosmetic impairment. In the majority of studies, repair is performed as part of abdominoplasty or ventral hernia repair [4–6].

In order to identify predictive factors for successful surgery, Strigård et al. correlated preoperative Ventral Hernia Pain Questionnaire scores with postoperative improvement in abdominal muscle strength. Patients were predominantly female (55/57) with median age of 43 years and median BMI of 23 kg/m^2^. The study found that pain while being seated for longer than 30 min and pain limiting the ability to participate in sports are preoperative predictors for successful surgery [7]. In another study by the same group of authors, patients were randomized between surgery and a training program supervised by a physiotherapist. Surgery resulted in significantly improved abdominal wall function compared to patients in the non-operative group [8].

Because the rectus abdominis muscles help stabilize the spine, researchers were interested in finding an association between diastasis recti and low back pain [9, 10]. After conducting a systematic review, Benjamin et al. concluded that there was a small association between diastasis recti and pelvic organ prolapse, and that diastasis recti may be associated with impaired health-related quality of life, impaired abdominal muscle strength and low back pain severity [10]. Wether there is an underlying connective tissue problem to explain the association remains unanswered.

**Surgical technique**

Surgical options for treatment of diastasis recti include linea alba plication, modified hernia repair techniques, minimally invasive techniques (endoscopic or laparoscopic), or a combination of open and minimally invasive (hybrid) techniques. A systematic review by Mommers et al. included 1591 patients and failed to demonstrate a difference in outcomes among different surgical approaches [4]. Another two systematic reviews showed high patient satisfaction after surgery, but no difference between the different surgical options [5, 6].

The largest randomized-controlled trial on diastasis recti by Emanuelsson et al. included 89 patients with diastasis > 3 cm [8]. Patients were randomized into three groups—plication in two layers using 2-0 barbed polydioxanone, repair with retromuscular polypropylene mesh or a supervised exercise program. The majority of patients (87/89) were female, with a median number of 2 prior pregnancies and a median BMI of 23. All patients were symptomatic and non-smokers. The study found no difference between the two surgical arms at 1 year follow-up, including improvement in abdominal wall function, quality of life, and complication rates.

**Choice of suture**

Gama et al. conducted a randomized-controlled trial comparing linea alba plication in two layers versus one layer using non-absorbable suture (nylon). The study included 30 patients aged 25–50 years with a BMI 18–30 kg/m^2^. Patients with pre-existing hernia, significant comorbidities or smoking were excluded. The study found no difference in outcomes and a shorter operating time for the single-layer plication (35 vs. 15 min). Of note, there was an unacceptably high recurrence rate of 33% in patients in whom a barbed suture was used [11]. Another randomized study compared non-absorbable (nylon) with absorbable (polydioxanone) sutures for plication and found no difference in recurrence at 6 months. The study was limited by a small sample size—10 patients in each group—and the fact that it was sponsored by the company producing the absorbable sutures [12]. Mestak et al. performed a linea alba plication in 51 patients using running, locked, #0 loop polydioxanone. In a case–control study using ultrasound, they compared the postoperative inter-recti distance between the study group and a control group of normal subjects. There was no difference between the inter-recti distances of the study group and healthy subjects at 21 months follow-up [13].

Minimally invasive techniques

In patients where excess skin removal and waist line definition are not needed, minimally invasive techniques become attractive. Endoscopic subcutaneous, laparoscopic, robotic, and hybrid approaches have been described as feasible and esthetically superior [14–20]. Traditionally, these approaches have been applied to diastasis associated with a hernia and often these repairs use mesh with or without the need for a fascial release. Endoscopic-assisted linea alba reconstruction (ELAR) is a hybrid approach where subcutaneous dissection is utilized to expose the anterior rectus sheath [17]. The anterior rectus sheath is released bilaterally and re-approximated to recreate the linea alba followed by augmentation with an onlay mesh. The endoscopic mini/less open sublay (EMILOS) technique is a hybrid procedure where the retromuscular space is dissected, the line alba plicated followed by placement of a large mesh in the retromuscular space as in a Rives-Stoppa repair [18]. The enhanced view totally extraperitoneal Rives-Stoppa (eTEP-RS) repair can be performed laparoscopically or robotically and, similar to the EMILOS approach, involves dissection of the retromuscular space, plication of line alba, and placement of a mesh [19]. Totally endoscopic subcutaneous dissection has also been described utilizing 3 suprapubic trocars. Following dissection, the linea alba is recreated with or without placement of an onlay mesh. [20]

However, there are no prospective studies comparing open and minimally invasive techniques. Published systematic reviews report no difference in outcomes, but are limited by low-quality data and high heterogeneity [4–6]. Since the indication for surgery in the majority of patients is cosmesis, a minimally invasive approach is the logical evolution in the surgical management of this condition when excess skin removal is not required.

**References** (level of evidence in parentheses)

1. Sperstad JB, Tennfjord MK2, Hilde G3, Ellström-Engh M4, Bø K (2016) Diastasis recti abdominis during pregnancy and 12 months after childbirth: prevalence, risk factors and report of lumbopelvic pain. Br J Sports Med. 50(17):1092–1096. 10.1136/bjsports-2016-096065. (**IV**)
2. Beer GM, Schuster A, Seifert B, Manestar M, Mihic-Probst D, Weber SA (2009)The normal width of the linea alba in nulliparous women. Clin Anat. 22(6):706–711. 10.1002/ca.20836. (**IV**)
3. Nahas FX. (2001) An aesthetic classification of the abdomen based on the myoaponeurotic layer. Plast Reconstr Surg 108(6):1787–1795. 10.1097/00006534-200111000-00057. (**IV**)
4. Mommers EHH, Ponten JEH, Al Omar AK, de Vries Reilingh TS, Bouvy ND, Nienhuijs SW. (2017) The general surgeon’s perspective of rectus diastasis. A systematic review of treatment options. Surg Endosc Jun 8. 10.1007/s00464-017-5607-9. [Epub ahead of print] (**IIA**)
5. Akram J, Matzen SH. (2013) Rectus abdominis diastasis. J Plast Surg Hand Surg 48(3):163-9. 10.3109/2000656x.2013.859145. Epub 2013 Nov 21. (**IIA**)
6. Hickey F, Finch JG, Khanna A (2011) A systematic review on the outcomes of correction of diastasis of the recti. Hernia 15(6):607-14. 10.1007/s10029-011-0839-4. Epub 2011 Jun 18. (**IIA**)
7. Strigård K, Clay L, Stark B, Gunnarsson U. (2017) Predictive Factors in the Outcome of Surgical Repair of Abdominal Rectus Diastasis. Plast Reconstr Surg Glob Open 5;4(5):e702. 10.1097/gox.0000000000000688. eCollection 2016 May. (**IV**)
8. Emanuelsson P, Gunnarsson U, Dahlstrand U, Strigård K, Stark B. (2016) Operative correction of abdominal rectus diastasis (ARD) reduces pain and improves abdominal wall muscle strength: A randomized, prospective trial comparing retromuscular mesh repair to double-row, self-retaining sutures. Surgery 160(5):1367–1375. 10.1016/j.surg.2016.05.035. Epub 2016 Jul 27. (**IB**)
9. Doubkova L, Andel R, Palascakova-Springrova I, Kolar P, Kriz J, Ko-besova A (2018) Diastasis of rectus abdominis muscles in low back pain patients. J Back Musculoskelet Rehabil 31(1):107–112. 10.3233/bmr-169687. (**IV**)
10. Benjamin DR, Frawley HC, Shields N, van de Water ATM, Taylor NF (2018) Relationship between diastasis of the rectus abdominis muscle (DRAM) and musculoskeletal dysfunctions, pain and quality of life: a systematic review. Physiotherapy pii: S0031-9406(18)30132-9. 10.1016/j.physio.2018.07.002. [Epub ahead of print] (**IIA**)
11. Gama LJM, Barbosa MVJ, Czapkowski A, Ajzen S, Ferreira LM, Nahas FX (2017) Single-Layer Plication for Repair of Diastasis Recti: The Most Rapid and Efficient Technique. Aesthet Surg J 37(6):698–705. 10.1093/asj/sjw263. (**IIB**)
12. Nahas FX, Augusto SM, Ghelfond C. (2001) Nylon versus polydioxa-none in the correction of rectus diastasis. Plast Reconstr Surg 107(3):700–716. (**IIB**)
13. Mestak O, Kullac R, Mestak J, Nosek A, Krajcova A, Sukop A. (2012) Evaluation of the long-term stability of sheath plication using absorbable sutures in 51 patients with diastasis of the recti muscles: an ultraso-nographic study. Plast Reconstr Surg 130(5):714e–719e.10.1097/prs.0b013e318267d806. (**III**)
14. Chang CJ. (2013) Assessment of videoendoscopy-assisted abdomi-noplasty for diastasis recti patients. Biomed J 36(5):252–256. 10.4103/2319-4170.113374. (**IV**)
15. Palanivelu C, Rangarajan M, Jategaonkar PA, Amar V, Gokul KS, Sri-kanth B. (2009) Laparoscopic repair of diastasis recti using the ‘Venetian blinds’ technique of plication with prosthetic reinforcement: a retrospective study. Hernia 13(3):287–292. 10.1007/s10029-008-0464-z. Epub 2009 Feb 12. (**IV**)
16. Shirah BH, Shirah HA (2016) The Effectiveness of Polypropylene Mesh in the Open and Laparoscopic Repair of Divarication of the Recti. J Med Imp Surg 1:105. 10.4172/jmis.1000105 (**IV**)
17. Köckerling F, Botsinis MD, Rohde C, Reinpold W, Schug-Pass C. (2017) Endoscopic-assisted linea alba reconstruction: New technique for treatment of symptomatic umbilical, trocar, and/or epigastric hernias with concomitant rectus abdominis diastasis. European Surgery 49(2):71–75. 10.1007/s10353-017-0473-1. (**III**)
18. Schwarz J, Reinpold W, Bittner R. (2017) Endoscopic mini/less open sublay technique (EMILOS)-a new technique for ventral hernia repair. Langenbecks Arch Surg 402(1):173–180. 10.1007/s00423-016-1522-0. Epub 2016 Oct 20. (**III**)
19. Belyansky I, Daes J, Radu VG, Balasubramanian R, Reza Zahiri H, Weltz AS, Sibia US, Park A, Novitsky Y (2018) A novel approach using the enhanced-view totally extraperitoneal (eTEP) technique for laparoscopic retromuscular hernia repair. Surg Endosc 32(3):1525–1532. (**III**)
20. Bellido Luque J1, Bellido Luque A, Valdivia J, Suarez Gráu JM, Gomez Menchero J, García Moreno J, Guadalajara Jurado J (2015) Totally endoscopic surgery on diastasis recti associated with midline hernias. The advantages of a minimally invasive approach. Prospective cohort study. Hernia 19(3):493–501 (**IV**)

## Chapter 3. Component separation techniques

### Frederik Berrevoet, Lars Nannestad Jorgensen

**Background**

Large ventral and incisional hernias, sometimes with loss of domain, remain a surgical challenge. Due to their relative rarity there is no exact estimate of their incidence. In 1951 Albanese et al. designed a first model of component separation of the abdominal wall, later elegantly refined by Ramirez in 1990 as a part of a study on human cadavers [1, 2]. The latter’s initial results showed the possibility of translating the abdominal midline on average 10 cm per side at the umbilical level when releasing the external oblique muscle. Component separation has been applied increasingly and modifications trying to tackle the main issues of the technique have been made. Described limitations of this technique are complications involving the skin and subcutaneous tissue, most likely caused by surgical interruption of perforating vessels during exposure of the oblique muscle [3]. To date, most commonly used techniques for releasing the various fascial components of the abdominal wall are the ‘open anterior approach’, the ‘transversus abdominis release’ (TAR), the ‘endoscopic anterior release’, and the ‘open anterior perforator preserving approach’ with their original description in the noted references [2, 4–6].

As the component separation techniques (CST) were not included in the former IEHS guidelines, a full literature search was performed.

**Key question**

1. When is any type of component separation indicated?

**Search terms**

The following search terms were used: ((component* separation) OR (separation of components) OR (myofascial release)) AND (hernia OR (abdominal wall) OR (”Hernia, Ventral”[Mesh])) AND (indication OR use).

**Search machines**

PubMed, Medline, and the Cochrane Library as well as Google scholar were searched for relevant studies.

Abstracts of resulted articles were reviewed for their relevance to component separation techniques and indications. In total 475 papers were analyzed, of which none specifically studied indications for CST.

**Indications for CST**


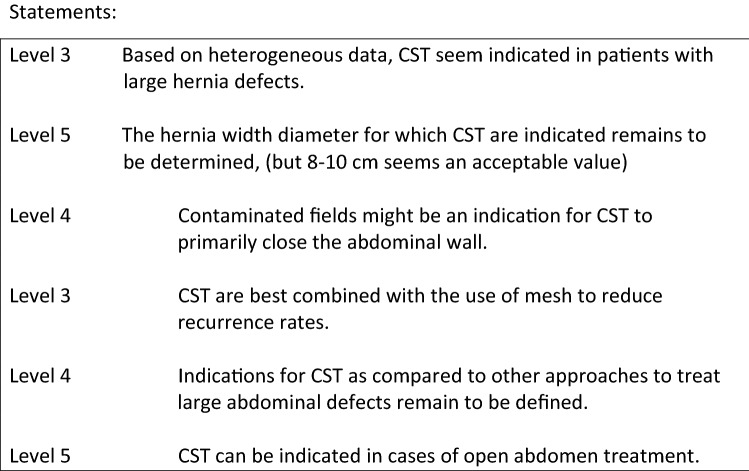



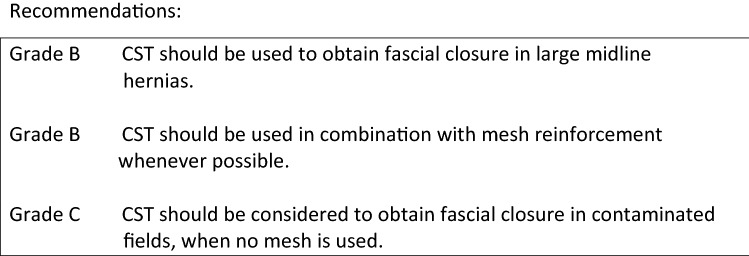


Current literature on CST is heterogeneous in various aspects: indications for use, mesh augmentation versus primary fascial closure using CST, different surgical techniques, and different abdominal wall components to be separated. No well-designed randomized-controlled trials are available for most indications and comparative studies between CST other popular techniques to treat giant hernias with or without loss of domain, like the use of botoxulin A and progressive pneumoperitoneum are also non-existing. The majority of reported cases for CST involve large ventral hernia defects in which primary closure of the fascial edges is not possible and for which CST seems more efficient than bridging of the defect with mesh [8]. However, the definition of ‘large’ defects also varies considerably among publications. Slater et al. defined a large defect as a hernia width of at least 10 cm [9], but failure of primary closure of the fascia might occur with smaller defect as well, mostly due to fibrosis, edema, or obesity.

Among others, de Vries-Reilingh et al. and Hodgkinson et al. showed that CST might facilitate primary fascial closure in patients with contamination of the abdominal cavity, especially in case no mesh will be used [10, 11]. However, the same Dutch group of de Vries-Reilingh observed a high recurrence rate after CST without the use of mesh, which was confirmed by other groups [12, 13].

Another possible indication for the use of CST might be the open abdomen patient. Although the number of patients reported is very low, CST might increase the fascial closure rate in this specific subset of patients (refs).

**References** (in parentheses the level of evidence)

1. Albanese AR (1951) [Gigantic median xipho-umbilical eventration; method for treatment]. Rev Asoc Med Argent.; 65(709-710):376–378 (**4**).
2. Ramirez OM, Ruas E, Dellon AL (1990) “Components separation” method for closure of abdominal-wall defects: an anatomic and clinical study. Plastic and reconstructive surgery 86(3):519–526 (**4**).
3. Lowe JB, Garza JR, Bowman JL, Rohrich RJ, Strodel WE (2000) Endoscopically assisted “components separation” for closure of abdominal wall defects. Plastic and reconstructive surgery 105(2):720–729 (**4**).
4. Novitsky YW, Elliott HL, Orenstein SB, Rosen MJ (2012) Transversus abdominis muscle release: a novel approach to posterior component separation during complex abdominal wall reconstruction. Am J Surg 204(5):709–716 (**4**).
5. Rosen MJ, Jin J, McGee MF, Williams C, Marks J, Ponsky JL (2007) Laparoscopic component separation in the single-stage treatment of infected abdominal wall prosthetic removal. Hernia 11(5):435–440 (**3**).
6. Saulis AS, Dumanian GA (2002) Periumbilical rectus abdominis perforator preservation significantly reduces superficial wound complications in “separation of parts” hernia repairs. Plast Reconst Surg. 109(7):2275–2280 (**3**).
7. Razavi SA, Desai KA, Hart AM, Thompson PW, Losken A (2018) The Impact of Mesh Reinforcement with Components Separation for Abdominal Wall Reconstruction. Am Surg 84(6):959–962 (**3**).
8. Holihan JL, Askenasy EP, Greenberg JA, Keith JN, Martindale RG, Roth JS, Mo J, Ko TC, Kao LS, Liang MK (2016) Ventral hernia outcome collaboration writing group. component separation vs. bridged repair for large ventral hernias: a multi-institutional risk-adjusted comparison, systematic review, and meta-analysis. Surg Infect (Larchmt) 17(1):17–26. 10.1089/sur.2015.124. Epub 2015 Sep 16 (**1A**).
9. Slater NJ, Montgomery A, Berrevoet F, Carbonell AM, Chang A, Franklin M, Kercher KW, Lammers BJ, Parra-Davilla E, Roll S, Towfigh S, van Geffen E, Conze J, van Goor H (2014) Criteria for definition of a complex abdominal wall hernia. Hernia 18(1):7–17. 10.1007/s10029-013-1168-6. Epub 2013 Oct 23 (**1A**).
10. de Vries Reilingh TS, van Goor H, Rosman C, Bemelmans MH, de Jong D, van Nieuwenhoven EJ, van Engeland MI, Bleichrodt RP (2003) “Components separation technique” for the repair of large abdominal wall hernias. J Am Coll Surg 196(1):32–37 (**3**).
11. Hodgkinson JD, Maeda Y, Leo CA, Warusavitarne J, Vaizey CJ (2017) Complex abdominal wall reconstruction in the setting of active infection and contamination: a systematic review of hernia and fistula recurrence rates. Colorectal Dis 19(4):319–330. 10.1111/codi.13609 (**1A**).
12. de Vries Reilingh TS, van Goor H, Charbon JA, Rosman C, Hesselink EJ, van der Wilt GJ, Bleichrodt RP (2007) Repair of giant midline abdominal wall hernias: “components separation technique” versus prosthetic repair: interim analysis of a randomized controlled trial. World J Surg 31(4):756–763 (**3**).
13. Harth KC, Rosen MJ (2010) Endoscopic versus open component separation in complex abdominal wall reconstruction. Am J Surg 199:342–347. 10.1016/j.amjsurg. 2009.09.015 (**3**).
14. Rasilainen SK, Mentula PJ, Leppäniemi AK (2016) Components separation technique is feasible for assisting delayed primary fascial closure of open abdomen. Scand J Surg 105(1):17–21 (**4**).
15. Sriussadaporn S, Sriussadaporn S, Pak-Art R, Kritayakirana K, Prichayudh S, Samorn P (2013) Management of difficult abdominal wall problems by components separation methods: a preliminary study in Thailand. J Med Assoc Thai 96(11):1449–1462 (**4**).
  1. **Key question**

Which type of anterior CST is preferred?

**Search terms**

The following search terms were used: ((component* separation) OR (separation of components) OR (myofascial release)) AND (hernia OR (abdominal wall) OR (”Hernia, Ventral”[Mesh])) AND (indication OR use).

**Search machines**

PubMed, Medline, and the Cochrane Library as well as Google scholar were searched for relevant studies.

Abstracts of resulted articles were reviewed for their relevance to the different component separation techniques. From the total of 475 papers extracted, 7 reviews [1–7] and 12 case–control studies [8–19] were applicable to the key question.


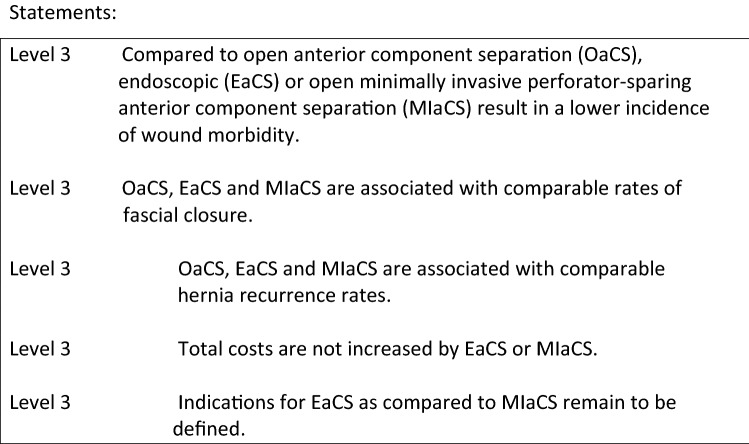



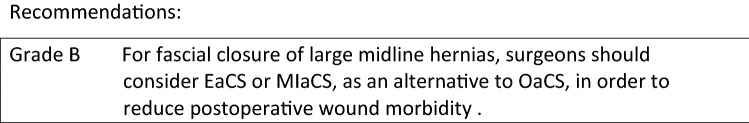


Various systematic reviews and meta-analyses have recently been reported to compare the different techniques of CST. The minimal invasive and endoscopic component separation have been suggested to reduce the postoperative wound morbidity as large subcutaneous dissection and skin flaps might be avoided achieving the same outcomes in terms of fascial closure rate.

In 2014 Feretis et al. reported 13 studies in their meta-analysis including 220 patients [3]. Overall they analyzed a wound complication rate of 17.5% versus 28% for endoscopic CST and Minimally Invasive CST respectively, resulting in a overall rate of 19.2%. However, when they only analyzed comparative studies, only 2 out of 5 studies [10, 12] showed a significantly lower incidence of wound morbidity in endoscopic and minimally invasive CST compared to open techniques, and the trend was clear in all studies [11, 13, 15]. This was confirmed by the systematic review of Switzer et al. who studied 63 studies including over 3000 patients with a wound complication rate of 34.6% for open versus 20.6% for endoscopic or minimally invasive techniques [2]. Jensen et al., in their review, only looked at studies that used the endoscopic CST as described by Rosen in 2007 [20] comparing it with the classical open Ramirez’ technique [21]. In total 5 retrospective cohort studies were observed with 163 patients, but again 43% versus 18% of wound morbidity was seen, in favor of the endoscopic CST [1].

Cornette et al., although analyzing the various anterior CST versus the posterior CST (TAR), found 13 studies for laparoscopic/endoscopic CST, only 5 on minimally invasive perforator sparing techniques and 22 on open CST. Considering wound morbidity, a slight trend in favor of perforator sparing techniques was found, versus open and laparoscopic techniques (16% vs. 21.4% and 20.3% respectively). However, as this trend is consistent throughout all the available data, despite their moderate to low quality, surgeons should consider endoscopic or minimally invasive (perforator sparing) CST, as an alternative to open CST, in order to reduce postoperative wound morbidity. Regarding recurrence rates after these various modalities, no differences are observed in the current medical literature. Furthermore, it is difficult to draw any conclusions regarding this parameter, as many variables influence recurrence rate in these large hernias.

**References** (in parentheses the level of evidence)

1. Jensen KK, Henriksen NA, Jorgensen LN (2014) Endoscopic component separation for ventral hernia causes fewer wound complications compared to open components separation: a systematic review and meta-analysis. Surg Endosc 28:3046–3052 (**1A**).
2. Switzer NJ, Dykstra MA, Gill RS, Lim S, Lester E, de Gara C, Shi X, Birch DW, Karmali S (2015) Endoscopic versus open component separation: systematic review and meta-analysis. Surg Endosc 29:787–795 (**1A**).
3. Feretis M, Orchard P (2015) Minimally invasive component separation techniques in complex ventral abdominal hernia repair: a systematic review of the literature. Surg Laparosc Endosc Percutan Tech 25:100–105 (**1A**).
4. Holihan JL, Askenasy EP, Greenberg JA, Keith JN, Martindale RG, Roth JS, Mo J, Ko TC, Kao LS, Liang MK (2016) Ventral Hernia Outcome Collaboration Writing Group. Component separation vs. bridged repair for large ventral hernias: a multi-institutional risk-adjusted comparison, systematic review, and meta-analysis. Surg Infect (Larchmt) 17:17–26 (**1A**).
5. Cornette B, De Bacquer D, Berrevoet F (2018) Component separation technique for giant incisional hernia: a systematic review. Am J Surg 215(4):719–726. 10.1016/j.amjsurg.2017.07.032. Epub 2017 Aug 10 (**1A**).
6. Scheuerlein H, Thiessen A, Schug-Pass C, Köckerling F (2018) What do we know about component separation techniques for abdominal wall hernia repair? Front Surg. 2018 (**5**).
7. Muse TO, Zwischenberger BA, Miller MT, Borman DA, Davenport DL, Roth JS. Outcomes after ventral hernia repair using the Rives-stoppa, endoscopic, and open component separation techniques. Am Surg. 2018 84(3):433–437 (**3**).
8. Albright E, Diaz D, Davenport D, Roth JS (2011) The component separation technique for hernia repair: a comparison of open and endoscopic techniques. Am Surg 77:839–43 (**3**).
9. Azoury SC et al. (2014) A single institutional comparison of endoscopic and open abdominal component separation. Surg Endosc 28:3349–58 (**3**).
10. Fox M, Cannon RM, Egger M, Spate K, Kehdy FJ (2013) Laparoscopic component separation reduces postoperative wound complications but does not alter recurrence rates in complex hernia repairs. Am J Surg 206:869–874 (**3**).
11. Giurgius M, Bendure L, Davenport DL, Roth JS (2012) The endoscopic component separation technique for hernia repair results in reduced morbidity compared to the open component separation technique. Hernia 16:47–51 (**3**).
12. Harth KC, Rosen MJ (2010) Endoscopic versus open component separation in complex abdominal wall reconstruction. Am J Surg 199:342–346 (**3**).
13. Lowe JB, Garza JR, Bowman JL, Rohrich RJ, Strodel WE (2000) Endoscopically assisted “components separation” for closure of abdominal wall defects. Plast Reconstr Surg 105:720–729 (**4**).
14. Parker M, Bray JM, Pfluke JM, Asbun HJ, Smith CD, Bowers SP (2011) Preliminary experience and development of an algorithm for the optimal use of the laparoscopic component separation technique for myofascial advancement during ventral incisional hernia repair. J Laparoendosc Adv Surg Tech A 21:405–410 (**4**).
15. Ghali S, Turza KC, Baumann DP, Butler CE (2012) Minimally invasive component separation results in fewer wound-healing complications than open component separation for large ventral hernia repairs. J Am Coll Surg 214:981–989 (**3**).
16. Saulis AS, Dumanian GA (2002) Periumbilical rectus abdominis perforator preservation significantly reduces superficial wound complications in “separation of parts” hernia repairs. Plastic Reconstr Surg 109:2275–2280 (**4**).
17. Patel KM, Nahabedian MY, Gatti M, Bhanot P (2012) Indications and outcomes following complex abdominal reconstruction with component separation combined with porcine acellular dermal matrix reinforcement. Ann Plastic Surg 69:394–398 (**4**).
18. Clarke JM (2010) Incisional hernia repair by fascial component separation: results in 128 cases and evolution of technique. Am J Surg 200:2–8 (**4**).
19. Butler CE, Campbell KT (2011) Minimally invasive component separation with inlay bioprosthetic mesh (MICSIB) for complex abdominal wall reconstruction. Plastic Reconstr Surg 128:698–709 (4).
20. Rosen MJ, Jin J, McGee MF, Williams C, Marks J, Ponsky JL (2007) Laparoscopic component separation in the single-stage treatment of infected abdominal wall prosthetic removal. Hernia 11:435–440 (4).
21. Ramirez OM, Ruas E, Dellon AL (1990) ‘‘Components separation’’ method for closure of abdominal-wall defects: an anatomic and clinical study. Plast Reconstr Surg 86:519–526 (4).
  2. **Key question**

Is a Transversus Abdominis Release (TAR) preferred over an anterior component separation technique?

**Search terms**

The following search terms were used: ((component* separation) OR (separation of components) OR (myofascial release)) AND (hernia OR (abdominal wall) OR (”Hernia, Ventral”[Mesh])) AND (anterior OR posterior).

**Search machines**

PubMed, Medline, and the Cochrane Library as well as Google scholar were searched for relevant studies.

Abstracts of resulted articles were reviewed for their relevance to the different component separation techniques. From the total of 106 papers extracted, 3 reviews [1–3] and 3 case–control studies [4–5] were applicable to the key question.


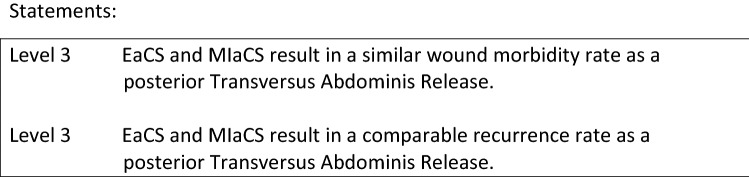



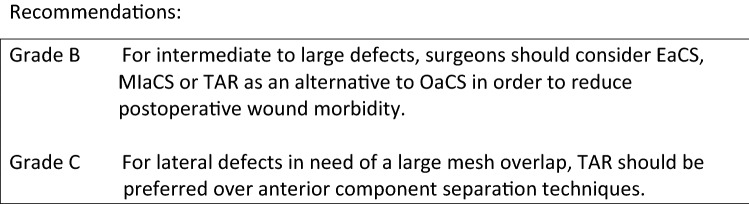


As a retromuscular mesh position in incisional hernia is still preferred over an onlay or intraperitoneal mesh repair, larger defects might be difficult to treat using this technique as the mesh overlap is limited by the lateral borders of the rectus sheet. Since the introduction of the posterior component separation technique using a transverses abdominis release (TAR), described by Novitsky et al., there is no need for large subcutaneous dissection to perform CST, and an extended wide overlap can be achieved for large defects and defects located lateral to the rectus muscles. Most of the literature involves retrospective observational cohort studies and not much comparative data are available. Recently, Hodgkinson et al. investigated the outcomes of posterior component separation and TAR with the open anterior CST. They report on 7 studies describing 281 cases of TAR for midline incisional hernia using a retromuscular mesh placement and compared those to 6 comparable studies describing 285 cases of open anterior CST and retromuscular mesh placement. Comparative analysis demonstrated no significant difference between hernia recurrence rate (*p* = 0.23) and no significant difference was found in wound complication rates between TAR and the open CST (*p* = 0.53). This finding was reported earlier already by Cornette et al. [2] and again confirmed recently by Scheuerlein et al. [3].

Therefore, surgeons are recommended to consider endoscopic, minimally invasive, or TAR as an alternative to OaCS in order to reduce postoperative wound morbidity. For lateral defects which may need a large mesh overlap, TAR should be preferred over anterior component separation techniques.

**References** (in parentheses the level of evidence)

1. Hodgkinson JD, Leo CA, Maeda Y, Bassett P, Oke SM, Vaizey CJ, Warusavitarne J (2018) A meta-analysis comparing open anterior component separation with posterior component separation and transversus abdominis release in the repair of midline ventral hernias. Hernia 22(4):617–626. 10.1007/s10029-018-1757-5. Epub 2018 Mar 7 (**1A**).
2. Cornette B, De Bacquer D, Berrevoet F (2018) Component separation technique for giant incisional hernia: A systematic review. Am J Surg 15(4):719–726. 10.1016/j.amjsurg.2017.07.032. Epub 2017 Aug 10 (**1A**).
3. Scheuerlein H, Thiessen A, Schug-Pass C, Köckerling F (2018) What Do We Know About Component Separation Techniques for Abdominal Wall Hernia Repair? Front Surg 27;5:24. 10.3389/fsurg.2018.00024 (**5**).
4. Parent B, Horn D, Jacobson L, Petersen RP, Hinojosa M, Yates R, Wright AS, Louie O (2017) Wound morbidity in minimally invasive anterior component separation compared to transversus abdominis release. Plast Reconstr Surg 139:472–479. 10.1097/prs.0000000000002957 (**3**).
5. Krpata DM, Blatnik JA, Novitsky YW, Rosen MJ (2012) Posterior and open anterior components separations: a comparative analysis. Am J Surg 203:318–322. 10.1016/j.amjsurg. 2011.10.009. Epub 2012 Jan 12 (**3**).

## Chapter 4. Key question: In which patient group is a transversus abdominis release (TAR) indicated?

### G Woeste, A de Beaux

The main goal of ventral hernia repair is reconstruction of the midline and bringing the rectus muscle together.

The recurrence rate has shown to be significantly lower when a bridging of the gap with the used mesh can be avoided [1].

A restoration of the midline is beneficial both in terms of functional results and recurrence rate.

Whenever it is not possible to close the linea alba in midline ventral hernia repair, a component separation technique (CST) is indicated. The TAR technique can be used to achieve midline closure in most of the cases. With an advancement of 8 to 12 cm per side Novitsky et al. have reported a closure rate of 97.2% [2]. When a recurrent hernia occurs after an anterior component separation (aCS) has been performed TAR has been shown to be an option for abdominal wall reconstruction in these complex cases [3]. However, it is an import rule that aCS and TAR should never be performed simultaneously at the same side.

Apart from midline incisional hernias TAR can be used for the repair of lateral hernias (L1–4). A case series of hernias after kidney transplantation has been published with low morbidity and low recurrence rate [4].

Also a cohort of parastomal hernias has been successfully treated by stoma relocation and closure of the lateral hernia using TAR (5).


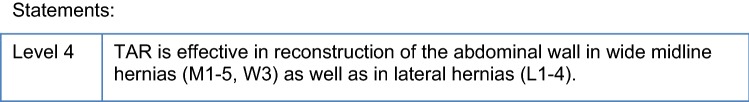



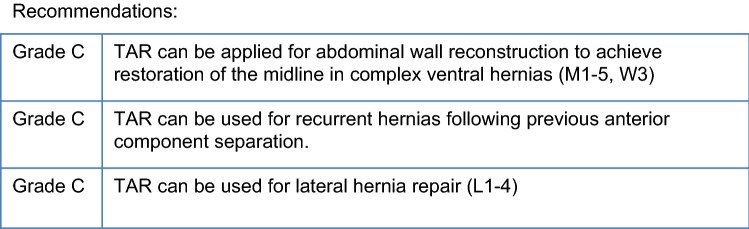


**References** (in parentheses the level of evidence)

1. Holihan JL, Nguyen DH, Nguyen MT, Mo J, Kao LS, Liang MK (2016) Mesh location in open ventral hernia repair: A systematic review and network Meta-analysis. World J Surg 40:89–99. (**1**)
2. Novitsky YW, Fayezizadeh M, Majumder A, Neupane R, Elliott HL, Orenstein SB (2016) Outcomes of posterior component separation with transversus abdominis muscle release and synthetic mesh sublay reinforcement. Ann Surg 264:226–232. (**3**)
3. Pauli EM, Wang J, Petro CC, Juza RM, Novitsky YW, Rosen MJ (2015) Posterior component separation with transversus abdominis release successfully addresses recurrent ventral hernias following anterior component separation. Hernia 19:285–291. (**l4**)
4. Petro CC, Orenstein SB, Criss CN, Sanchez EQ, Rosen MJ, Woodside KJ, Novitsky YW (2015) Transversus abdominis muscle release for repair of complex incisional hernias in kidney transplant recipients. Am J Surg 210:334–339. (4)
5. Raigani S, Criss CN, Petro CC, Prabhu AS, Novitsky YW, Rosen MJ (2014) Single-center experience with Parastomal hernia repair using retromuscular mesh placement. J Gastrointest Surg 18:1673–1677. (4)

**Should the TAR be done open or endoscopically?**

The open TAR technique has first been described by Novitsky in 42 patients with complex hernias with a mean defect size of 366 cm^2^ [1].

The largest series of open TAR (O-TAR) is published by the same author [2]. In this retrospective series of 428 patients the incidence of surgical site events was 18.7% with 9.1% SSI and no mesh removal. The recurrence rate after 1 year was only 3.7%. Winder et al. described similar results in their retrospective review of 37 patients with 5.4% SSI and 2.7% recurrences at 2 years only in patients where a midline closure could not have been achieved [3].

The laparoscopic technique of TAR has been published in 2016 with a cohort of 3 patients showing no complications [4].

Also a robotic (rTAR) approach has been described [5].

The published results of rTAR are very limited. A nationwide series of 6 patients in Brazil has been described [6]. The authors conclude that this technique is feasible with two postoperative complications requiring reoperation.

No randomized-controlled trials comparing open and laparoscopic or robotic TAR are published so far. Two retrospective studies compare the results of O-TAR and rTAR.

The Cleveland group compared 38 patients who underwent rTAR with a matched historic cohort of 76 O-TAR cases [7]. Comparing the patient characteristics, more ASA III patients were found in the O-TAR group. The robotic approach showed significant longer OR time (299 ± 95 vs. 211 ± 63, *p* < 0.001). The incidence of wound morbidity did not show any significant difference between the two techniques for both SSE and SSI. The rTAR group showed lower blood loss (49 ± 60 ml vs. 139 ± 149 ml, *p* < 0.001), less systemic complications (0 vs. 17.1%, *p* = 0.026), and a shorter hospital stay (1.3 ± 1.3 days vs. 6.0 ± 3.4 days, *p* < 0.001).

A retrospective review of 102 patients, 26 rTAR and 76 O-TAR, were compared by Bittner et al. [8]. Comparing the comorbidities, diabetes was more common in the O-TAR group (22.3% vs 0%, *p* = 0.01). The defect size was comparable (260 ± 209 cm^2^ vs. 235 ± 107 cm^2^, *p* = 0.55). In this cohort the OR time was significantly longer with rTAR (287 ± 121 vs. 365 ± 78 min, *p* < 0.01). The surgical site events were the same in both groups (6.6% vs. 7.7%, *p* = 1.0). There was no significant difference in morbidity. The length of hospital stay was shorter after rTAR (3 vs. 6 days).

**Table:** 

Level 3	Both open and minimally invasive TAR are safe procedures.
Level 3	rTAR is associated with longer operative time compared to O-TAR.
Level 3	rTAR can reduce postoperative length of hospital stay compared to O-TAR.
Level 3	O-TAR and rTAR show the same incidence of postoperative wound morbidity.

**Table:** 

Grade C	TAR can be performed open, laparoscopic and robotic

**References** (in parentheses the level of evidence)

1. Novitsky YW, Elliott HL, Orenstein SB, Rosen MJ (2012) Transversus abdominis muscle release: a novel approach to posterior component separation during complex abdominal wall reconstruction. Am J Surg 204:709–716. (**4**)
2. Novitsky YW, Fayezizadeh M, Majumder A, Neupane R, Elliott HL, Orenstein SB (2016) Outcomes of posterior component separation with transversus abdominis muscle release and synthetic mesh sublay reinforcement. Ann Surg 264:226–232. (**3**)
3. Winder JS, Behar BJ, Juza RM, Potochny J, Pauli EM (2016) Transversus abdominis release for abdominal wall reconstruction: early experience with a novel technique. J Am Coll Surg 223:271–278. (**4**)
4. Belyansky I, Zahiri HR, Park A (2016) Laparoscopic transversus abdominis release, a novel minimally invasive approach to complex abdominal wall reconstruction. Surg Innov 23: 134–141. (**5**)
5. Ballecer C, Parra-Davila E (2016) Robotic ventral hernia repair. In: Novitsky YW (ed) Hernia surgery, Springer, New York, pp 273–286. (**5**)
6. Amaral MVFD, Guimaraes JR, Volpe P, Oliveira FMM, Domene CE, Roll S, Cavazzola LT (2017) Robotic transversus abdominis release (TAR): is it possible to offer minimally invasive surgery for abdominal wall complex defects? Rev Col Bras Cir 44:216–219. (**5**)
7. Martin del Campo LA, Weltz AS, Belyansky I, Novitsky YW (2018) Comparative analysis of perioperative outcomes of robotic versus open transversus abdominis release. Surg Endosc 32:840–845. (**4**)
8. Bittner JG 4^th^, Alrefai S, Mabe M, Del Prado PAR, Clingempeel NL (2018) Comparative analysis of open and robotic transversus abdominis release for ventral hernia repair. Surg Endosc 32:727–734. (**4**)

## Chapter 5. Key Question: The role of preoperative adjunct interventions in ventral hernia repair

### H. Hoffmann, P. Kirchhoff, J. Kukleta, W. Reinpold

**Search items**

“Botox AND Hernia”, “Botulinum Toxin A AND Hernia”, “Component Separation AND Botulinum Toxin A”, Component Separation” AND “Hernia”, Progressive Pneumoperitoneum AND Hernia”

A systematic search of the available literature was performed in September 2017 of Medline, PubMed, Cochrane Library, and relevant journals and reference lists using the above-listed search terms. The search found 38 articles; however, only 26 studies were suitable for this review in terms of content.


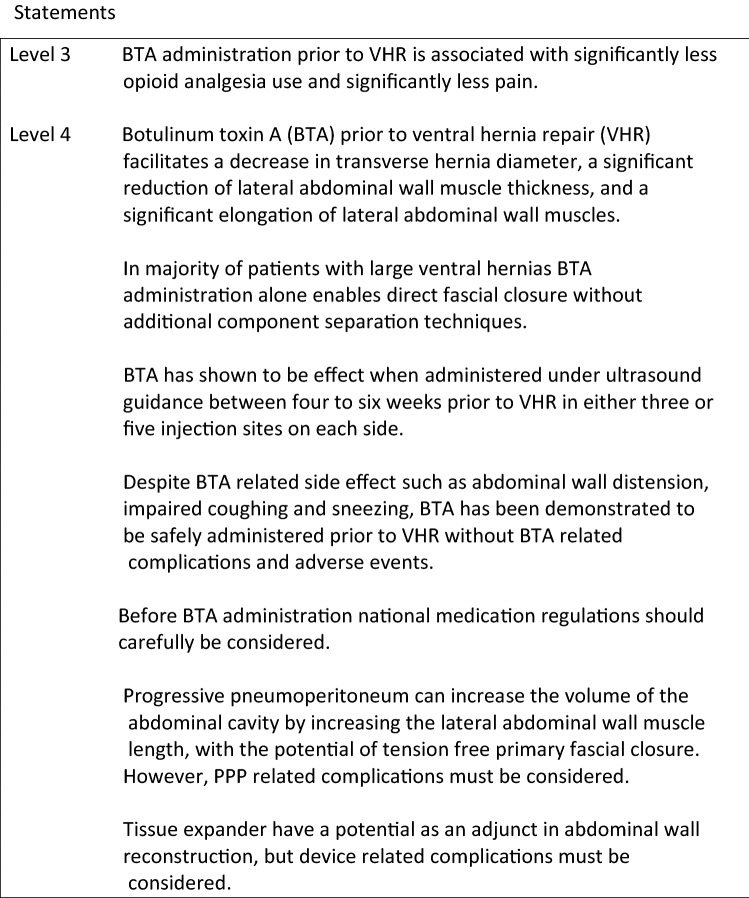



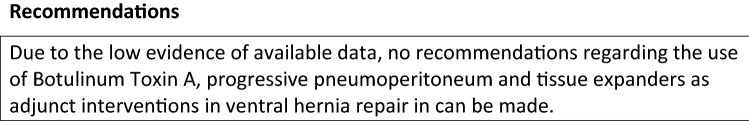


**Introduction**

Techniques such as progressive pneumoperitoneum (PPP), tissue expander (TE), and—most recently—Botulinum Toxin A (BTA) have gained some interest as an adjunct in the surgical approach of large incisional hernias, to gain primary fascial closure (PFC).

**Botulinum Toxin A**

Botulinum Toxin A (BTA) is a neurotoxic protein produced by clostridium botulinum. The paralyzing effect reaches a maximum 2 weeks after topical administration and declines gradually after 2–3 months [1–3]. Several clinical studies investigated the effect of BTA on abdominal wall muscle parameters using CT scans. Ibarra-Hurtado et al. [4] demonstrated a significant reduction of the transverse hernia defect in 12 patients. PFC was gained in six patients with BTA alone and with additional component separation (CS) in further six patients. In a subsequent study of 17 patients they demonstrated a significant reduction of lateral abdominal wall muscle thickness and the transverse hernia diameter, and a significant elongation of lateral abdominal wall muscles after BTA application [5]. PFC was possible in all patients, of which nine needed additional CS. Farooque et al. reported a significant increase in mean length of the lateral abdominal muscles post-BTA in a case series of 8 patients achieving PFC in all cases [6]. Also, Elstner et al. reported a significant increase in mean length of the lateral abdominal wall muscles in 27 patients, achieving PFC in all patients, with additional CS in six patients. Another study evaluated BTA administration in 56 consecutive patients undergoing VHR [7], of which 18 patients additionally underwent progressive pneumoperitoneum (PPP). They reported a significant increase in the lateral abdominal wall length. PFC was achieved in all patients, in 16% with additional CS. Another study from Elstner et al. investigated BTA and PPP administration in 16 patients with loss of domain undergoing VHR[8], achieving PFC in all patients without additional surgical dissection. Bueno-Lledó et al. performed a prospective observational study using BTA and PPP in 45 patients with loss of domain. They found a significant reduction of the VIH/VAC (volumes of the incisional hernia/volume of abdominal cavity) ratio by 14%. PFC was achieved in all patients with CS.

There are seven clinical studies reporting the effect of BTA administration alone regarding avoidance of additional CS techniques [4–7, 9–11]. In total 150 patients were enrolled in these studies, achieving PFC with BTA alone in 78% (*n* = 117). Considering that BTA reaches its maximum paralyzing effect after 2 weeks [1–3], timing of BTA application seems of somewhat importance. The available clinical studies follow different timing concepts ranging from the day of surgery up to 6 weeks prior to surgery [4–12]. Regarding supportive imaging guidance during BTA administration, majority of available studies report the use of ultrasound [5, 6, 8–12]. Only on study used musculature electromyography [4]. The injection volume, units, and concentration of BTA administration in VHR shows heterogeneity amongst available clinical studies. Three studies used 500 units of BTA in total [4, 5, 12]. Six studies used a total dose of 300 units BTA [6–11]. Regarding concentration and administered volume of BTA authors reported BTA concentration of 100 units/ml in 5 ml 0.9% saline solution [5], 10 units/ml in 50 ml in 0.9% saline solution [12] or 2 units/ml in 150 ml 0.9% saline solution [6–11]. Regarding injections sites, authors either perform three [6–11] or five [4, 5, 12] injections per side. Only one study investigated the effect of BTA administration on postoperative pain in a prospective cohort of 22 patients with BTA administration prior to VHR compared with a historic matched control group (*n* = 66). Patients with BTA administration had significantly less pain when compared to controls [10]. Since BTA is neurotoxic, potential complications or adverse events (AE) related to BTA application need to be addressed before injection. Only one study reported side effects of BTA administration such as abdominal wall distension, impaired coughing, and sneezing [11], while the majority of available clinical studies reported no BTA related adverse events or complications [5–12].

There are three reviews investigating the effect of BTA in incisional hernia repair including 15 studies with 259 patients [13], 6 studies with 133 patients [14] and 3 studies with 56 patients [15], respectively. The reviews reported PFC rates of 100% [13] and 84% [14] and decreases in the ventral hernia defect size [15]. Due to the small sample sizes and heterogeneity of included studies and the lack of standardization of BTA administration the level of evidence remained low (3a).

**Progressive pneumoperitoneum**

The concept of progressive pneumoperitoneum (PPP) was first described 1947 by Goni Moreno [16] and has been modified by other groups ever since. It consists of repeatedly inflating air into the abdominal cavity using sterile catheters over a few days, gaining an enlargement of the abdominal cavity to obtain hernia reduction and tension-free closure of large abdominal wall hernias. Since then a view case series and cohort studies with low number of patients have been published [13, 17–25]. All studies differ in terms of used gas for insufflation, patient population, timing, frequency, and volume of PPP insufflation. Investigated indications for PPP were giant incisional hernias [17–19, 23], large inguinal hernias [19] or patients with loss of domain [20–22, 24]. The timing of PPP insufflation showed differences among the available studies, starting at means between 5 and 15 days prior to surgery. PPP insufflation was repeated every day in some studies [19, 22, 23], other studies had longer insufflation intervals of 2 days or more [17, 18, 20, 21, 24]. The totally applied PPP volume ranged at means from 12 to 23 l with insufflation volumes of 1000-4000 ml per session. Adverse events (AE) were reported in most studies. Most frequent AE was shoulder pain in up to 24% [17, 18], limiting the amount of insufflated air. Bleeding complications [17, 18], catheter misplacement [17–19, 22], emphysema [19–21, 23], and catheter infection [22] have also been reported. However, PPP has been demonstrated to increase the volume of the abdominal cavity [17, 21, 24] by increasing the lateral abdominal wall muscle length [23]. As highlighted in a recent review [13], tension-free PFC after PPP in large ventral hernias was achieved in 84% of cases with a reported recurrence rate of 7.2%.

**Tissue expanders**

The purpose of tissue expanders (TE) is to stretch skin or the underlying fascia allowing PFC in larger hernias. TE can be positioned subcutaneously in cases of skin loss, intermuscular between the external and internal oblique muscles in cases of large ventral hernias and intra-abdominal in cases of congenital abdominal wall defects [26]. There is no consensus regarding indications, optimal technique, and TE associated risks. Recent reviews with large heterogeneity regarding study design, study population, number of patients, indication, and position of TE [13, 26] demonstrated PFC rates of 93% using TE.

**References** (in parentheses the level of evidence)

1. Nigam PK, Nigam A (2010) Botulinum toxin. Ind J Dermatol 55:8–14.
2. Truong D (Daniel D., Dressler D, Hallett M (2009) Manual of botulinum toxin therapy. Cambridge University Press
3. Hughes AJ (1994) Botulinum Toxin in Clinical Practice. Drugs 48:888–893.
4. Ibarra-Hurtado TR, Nuño-Guzmán CM, Echeagaray-Herrera JE, Robles-Vélez E, de Jesús González-Jaime J (2009) Use of Botulinum Toxin Type A Before Abdominal Wall Hernia Reconstruction. World J Surg 33:2553–2556. (**4**)
5. Ibarra-Hurtado TR, Nuno-Guzmán CM, Miranda-Diaz AG, Troyo-Sanromán R, Navarro-Ibarra R, Bravo-Cuellar L (2014) Effect of botulinum toxin type A in lateral abdominal wall muscles thickness and length of patients with midline incisional hernia secondary to open abdomen management. Hernia 18:647–652. (**4**)
6. Farooque F, Jacombs ASW, Roussos E, Read JW, Dardano AN, Edye M, Ibrahim N (2016) Preoperative abdominal muscle elongation with botulinum toxin A for complex incisional ventral hernia repair. ANZ J Surg 86:79–83. (**4**)
7. Rodriguez-Acevedo O, Elstner KE, Jacombs ASW, Read JW, Martins RT, Arduini F, Wehrhahm M, Craft C, Cosman PH, Dardano AN, Ibrahim N (2017) Preoperative Botulinum toxin A enabling defect closure and laparoscopic repair of complex ventral hernia. Surg Endosc 32:831–839. (**4**)
8. Elstner KE, Read JW, Rodriguez-Acevedo O, Ho-Shon K, Magnussen J, Ibrahim N (2017) Preoperative progressive pneumoperitoneum complementing chemical component relaxation in complex ventral hernia repair. Surg Endosc 31:1914–1922. (**4**)
9. Zielinski MD, Goussous N, Schiller HJ, Jenkins D, Ibarra-Hurtado TR, Nuño-Guzmán CM (2013) Chemical components separation with botulinum toxin A: A novel technique to improve primary fascial closure rates of the open abdomen. Hernia 17:101–107. (**4**)
10. Zendejas B, Khasawneh MA, Srvantstyan B, Jenkins DH, Schiller HJ, Zielinski MD (2013) Outcomes of Chemical Component Paralysis Using Botulinum Toxin for Incisional Hernia Repairs. World J Surg 37:2830–2837. (**3b**)
11. Elstner KE, Read JW, Rodriguez-Acevedo O, Cosman PH, Dardano AN, Jacombs ASW, Edye M, Zea A, Boesel T, Mikami DJ, Ibrahim N (2016) Preoperative chemical component relaxation using Botulinum toxin A: enabling laparoscopic repair of complex ventral hernia. Surg Endosc 31:1–8. (**4**)
12. Bueno-Lledó J, Torregrosa A, Ballester N, Carreño O, Carbonell F, Pastor PG, Pamies J, Cortés V, Bonafé S, Iserte J (2017) Preoperative progressive pneumoperitoneum and botulinum toxin type A in patients with large incisional hernia. Hernia 21:233–243. (**4**)
13. Alam NN, Narang SK, Pathak S, Daniels IR, Smart NJ (2016) Methods of abdominal wall expansion for repair of incisional herniae: a systematic review. Hernia 20:191–199. (**3a**)
14. Soltanizadeh S, Helgstrand F, Jorgensen LN (2017) Botulinum Toxin A as an Adjunct to Abdominal Wall Reconstruction for Incisional Hernia. Plast Reconstr Surg Glob Open 5:e1358. (**3a**)
15. Weissler JM, Lanni MA, Tecce MG, Carney MJ, Shubinets V, Fischer JP (2017) Chemical component separation: a systematic review and meta-analysis of botulinum toxin for management of ventral hernia. J Plast Surg Hand Surg 1–9. (**3a**)
16. Moreno IG (1947) Chronic eventrations and large hernias; preoperative treatment by progressive pneumoperitomeum; original procedure. Surgery 22:945–53. (**4**)
17. Caldironi MW, Romano M, Bozza F, Pluchinotta AM, Pelizzo MR, Toniato A, Ranzato R (1990) Progressive pneumoperitoneum in the management of giant incisional hernias: A study of 41 patients. Br J Surg 77:306–307. (**4**)
18. Toniato A, Pagetta C, Bernante P, Piotto A, Pelizzo MR (2002) Incisional hernia treatment with progressive pneumoperitoneum and retromuscular prosthetic hernioplasty. Langenbeck Arch Surg 387:246–248. (**4**)
19. Mayagoitia JC, Suárez D, Arenas JC, Díaz de León V (2006) Preoperative progressive pneumoperitoneum in patients with abdominal-wall hernias. Hernia 10:213–217. (**4**)
20. Sabbagh C, Dumont F, Robert B, Badaoui R, Verhaeghe P, Regimbeau JM (2011) Peritoneal volume is predictive of tension-free fascia closure of large incisional hernias with loss of domain: A prospective study. Hernia 15:559–565. (**4**)
21. Sabbagh C, Dumont F, Fuks D, Yzet T, Verhaeghe P, Regimbeau JM (2012) Progressive preoperative pneumoperitoneum preparation (the Goni Moreno protocol) prior to large incisional hernia surgery: Volumetric, respiratory and clinical impacts. A prospective study. Hernia 16:33–40. (**4**)
22. Alyami M, Passot G, Voiglio E, Lundberg PW, Valette PJ, Muller A, Caillot JL (2015) Feasibility of Catheter Placement under Ultrasound Guidance for Progressive Preoperative Pneumoperitoneum for Large Incisional Hernia with Loss of Domain. World J Surg 39:2878–2884. (**4**)
23. Dumont F, Fuks D, Verhaeghe P, Brehant O, Sabbagh C, Riboulot M, Yzet T, Regimbeau JM (2009) Progressive pneumoperitoneum increases the length of abdominal muscles. Hernia 13:183–187. (**4**)
24. Renard Y, Lardière-Deguelte S, de Mestier L, Appere F, Colosio A, Kianmanesh R, Palot JP (2016) Management of large incisional hernias with loss of domain: A prospective series of patients prepared by progressive preoperative pneumoperitoneum. Surgery 160:426–435. (**4**)
25. Cuminal L, Rousset P, Passot G, Caillot JL, Valette PJ, Muller A (2017) Image-guided preoperative progressive pneumoperitoneum for large incisional hernia repair. Diagn Interv Imaging 98:507–509. (**4**)
26. Wooten KE, Ozturk CN, Ozturk C, Laub P, Aronoff N, Gurunluoglu R (2017) Role of tissue expansion in abdominal wall reconstruction: A systematic evidence-based review. J Plast Reconstr Aesthet Surg 70:741–751. (**3a**)

## Chapter 6. Robotic ventral/incisional hernia repair

### Jeremy A Warren, Alfredo M Carbonell, Davide Lomanto, R. Fortelny, Hrishekesh P Salgaonkar, Kevin Bain, Vadim Meytes, George Ferzli

**Key question**

1. What are the differences between Robotic Intraperitoneal Onlay of Mesh (rIPOM) and standard IPOM?

The following search terms were used to identify the relevant literature on robotic ventral hernia repair in July 2018. Abstracts of resulted articles were reviewed for their relevance to robotic ventral hernia repair. “Robotic ventral hernia” search identified 60 articles, 26 of which were relevant after review of the abstract. “Robotic incisional hernia” resulted in a total of 59 articles, with 3 additional relevant studies identified. Six additional articles were found using “robotic TAR,” 2 of which were relevant and included. Search for “robotic transversus abdominis release” revealed no additional articles, nor did search for “robotic component separation.” Finally, “robotic abdominal wall reconstruction” identified 1 additional relevant publication included in this review.

**Introduction**

Though first described in 2003 [1], there has been relatively little interest in robotic surgery for hernia repair until the last several years. Of 32 relevant articles in the published literature, 19 have been published in 2017–2018 alone. Technological interest, surgeon ergonomics, improved 3-dimensional visualization, and articulating instruments that greatly facilitate intracorporeal suturing and dissection are among the leading reasons for the exponential growth in robotic hernia repair. Disadvantages of the robotic platform are the loss of tactile feedback, relying entirely on visual cues, and intimate knowledge of tissue handling [2]. Additionally, cost can be significant barrier to utilization and allocation of health care resources [2–4]. Durability of various robotic repair techniques remains an unanswered clinical outcome. No studies to date have significantly long enough follow-up to determine hernia recurrence rates or other potential long-term complications. Several techniques have been described using the robot, including standard intraperitoneal onlay of mesh (rIPOM) similar to that of standard laparoscopic ventral hernia repair with intraperitoneal mesh (LVHR), transabdominal preperitoneal repair (rTAPP), retromuscular repair with or without transversus abdominis release (rRVHR or rTAR), and most recently a retromuscular repair using an extended totally extraperitoneal (eTEP) approach.

**Robotic Intraperitoneal Onlay of Mesh (rIPOM)**


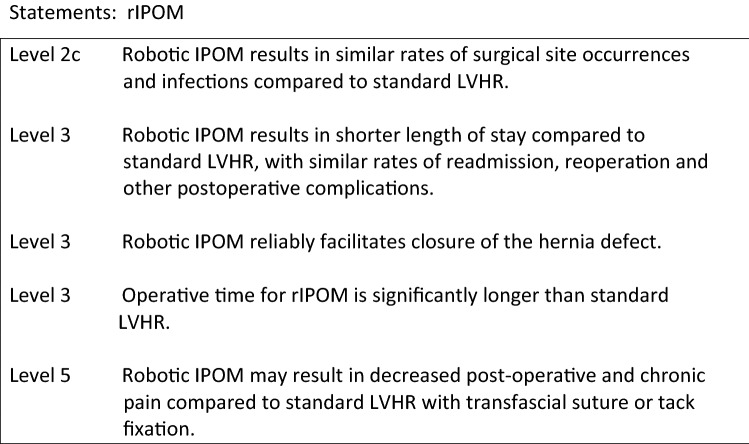



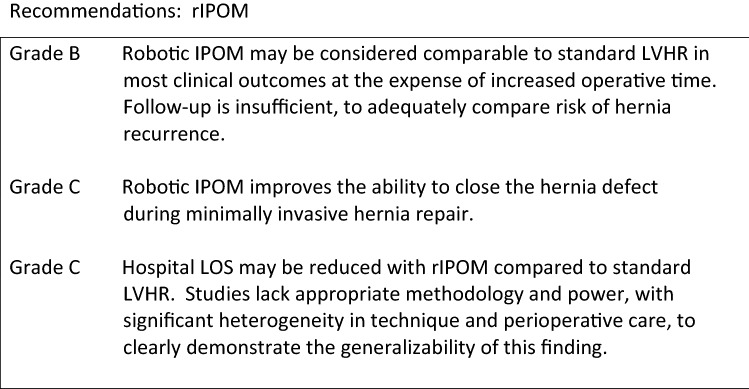


The majority of reported cases involve the use of the robot to perform a standard laparoscopic approach with placement of mesh in an intraperitoneal position, with the addition of standard closure of the defect. The primary benefit of this approach over LVHR is the ability to reliably close the hernia defect. The benefits of defect closure have been demonstrated in a number of studies, primarily in reducing the rate of seroma formation, and possibly reduction in hernia recurrence [5–10]. Abdominal wall tension required to close the hernia defect may be offset by additional transfascial suture fixation [11] or use of myofascial release [12, 13]. Comparative series of rIPOM vs LVHR indicate higher rates of fascial closure for robotic repair, but all are retrospective series without a protocolized approach to defect closure [8, 14–17].

Operative time is significantly longer with rIPOM compared to LVHR in all four comparative trials [8, 15–17]. This is likely associated with the differences in technique, primarily in defect closure and suture fixation of the mesh rather than tacks, and the learning curve for robotic repair. Intracorporeal suture fixation of the mesh, rather than standard tack or transabdominal wall sutures, is often touted to reduce postoperative and chronic pain compared to LVHR [2, 3, 18]. While there is some evidence that traditional fixation techniques, and the use of transfascial sutures in particular, may lead to greater postoperative pain [19–22], data are lacking to support this finding. There is, however, a consistently demonstrated reduction in hospital length of stay, which may be a surrogate marker for decreased early postoperative pain following rIPOM. The only comparative study evaluating pain as a specific secondary outcome showed no difference in narcotic use between LVHR and rRVHR/rTAR [14].

Rates of clinically significant wound complications are largely unaffected by rIPOM compared to LVHR. Rate of SSI is reported between 0.9 and 3.8%, with a single outlier study demonstrating a 9.1% rate of SSI (single patient in a case series of 11 patients) [2, 3, 12, 13, 18, 23, 24]. Surgical site occurrences are similarly low with rIPOM (0.9–3.8%) [2, 3, 12, 13, 18, 23, 24]. One study demonstrated a lower rate of SSO after rIPOM [17]. No differences were seen in patients requiring procedural intervention in the treatment of SSO or SSI in these studies. Overall complications were reduced for rVHR compared to LVHR after propensity score analysis from the New York State Planning and Research Cooperative System [25]. This study does not account for specific technique of RVHR, however. Similarly, evaluation of the National Inpatient Sample (NIS) demonstrates the safety and efficacy of rVHR, with similar rates of minor and major complications compared to LVHR [26]. Interrogation of the Vizient database demonstrated no benefit of rVHR over LVHR in clinical outcomes, with both procedures improving rates of readmission and LOS over OVHR [27]. This study also cannot account for specific surgical technique.

Hospital length of stay ranges from 0 to 2.5 days for rIPOM. Three of five comparative studies demonstrated a statistically significant difference in LOS [8, 14–16]. Prabhu et al. analyzed 186 rIPOM vs 452 LVHR in the Americas Hernia Society Quality Collaborative (AHSQC) database, demonstrating a reduction in LOS from 1 to 0 days with rIPOM (p < 0.001) [15]. Alteiri et al. similarly demonstrated a shorter LOS after RVHR [25]. Warren et al. demonstrated a shorter median LOS with rRVHR compared to IPOM LVHR [14]. No inferences can be drawn regarding hernia recurrence due to lack of long-term follow-up.

**Robotic Transabdominal Preperitoneal Repair (rTAPP)**


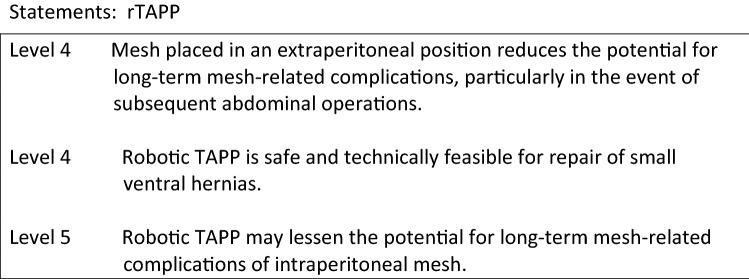



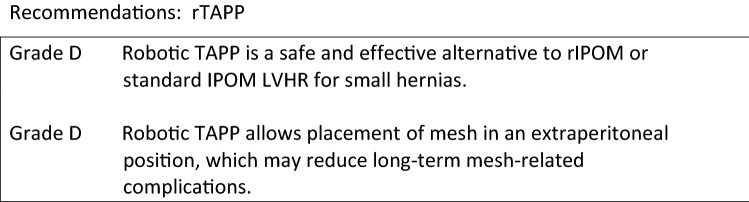


Placement of mesh in an extraperitoneal position may reduce long-term mesh-related complications compared to standard IPOM, particularly in the event of subsequent abdominal operations [28–33]. Laparoscopic TAPP repair of ventral hernia has been described, with favorable results [34, 35]. The delicate dissection of the peritoneum may be facilitated by robotic instrumentation. Three studies have been published to date examining the rTAPP approach. Sugiyama et al. used an rTAPP approach for repair of ventral hernia in three patients. Mean operative time was 164 min, with no reported operative or perioperative complications. Orthopoulos et al. reported rTAPP repair in 54 patients. Mean OR time was 73 min, with two reported complications; a seroma requiring percutaneous drainage and rectus sheath hematoma resulting in readmission and transfusion [36]. Finally, Kennedy et al. compared 27 rIPOM to 36 rTAPP patients. Operative time was similar between groups, as were minor perioperative complications [37]. No inferences can be drawn regarding hernia recurrence due to lack of long-term follow-up.

**Robotic retromuscular ventral hernia repair (rRVHR), robotic transversus abdominis release (rTAR), and robotic extended totally extraperitoneal (eTEP)**


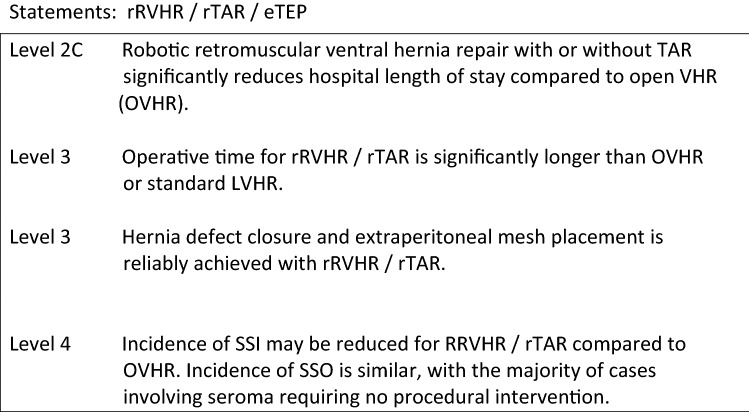



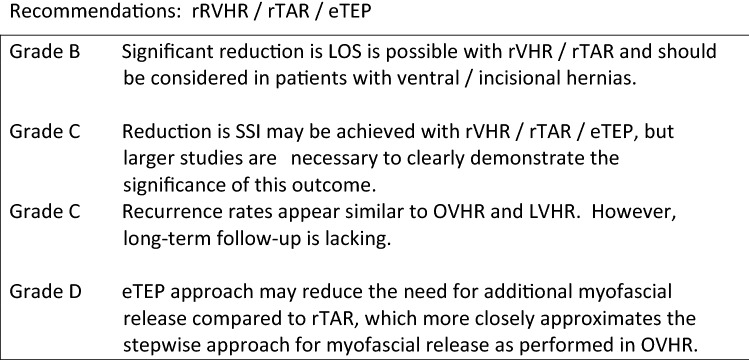


Perhaps the greatest promise for robotic hernia repair is the capability of this platform to duplicate an open retromuscular hernia repair, widely considered to be the standard for OVHR. The first description of this approach was reported in 2012 [38]. The potential advantage of this approach is the ability to utilize the robot to perform myofascial release of the rectus abdominis and/or transversus abdominis to facilitate medialization of the hernia defect for closure under reduced tension and placement of mesh in the retromuscular (extraperitoneal) space. The first study reporting outcomes of rRVHR/rTAR demonstrated a reduction in LOS compared to standard LVHR (1 vs. 2 days; *p* = 0.004), with similar rates of SSI, SSO requiring procedural intervention (SSOPI), and reliable closure of the hernia defect. Operative time was significantly longer with robotic repair [14]. However, this comparison involved two very different hernia techniques for hernia repair. Comparison of rRVHR/rTAR with open retromuscular repair is more appropriate. In the largest series to date, patients in the AHSQC undergoing rRVHR or rTAR were compared to those undergoing open retromuscular VHR. After propensity score matching, 111 rRVHR and 222 OVHR cases were identified. Groups were similar in hernia morphology and patient comorbidities. Robotic repair resulted in significantly shorter LOS compared to OVHR [39]. Two single-center comparative studies of rTAR to open TAR (O-TAR) were published this year. Martin del Campo et al. compared 38 rTAR to a matched cohort of 76 O-TAR, demonstrating a significant reduction in LOS from 6 to 1.3 days (*p* < 0.001) with rTAR, and reduction in both SSO and SSI, though this did not reach statistical significance. Bittner, et al., compared 26 rTAR to 76 O-TAR. No difference was seen in SSO or SSI, though LOS was again significantly shorter after rTAR than O-TAR [40]. Operative time was significantly longer for rRVHR/rTAR in each of these studies. No inferences can be drawn regarding hernia recurrence due to lack of long-term follow-up.

**Costs**


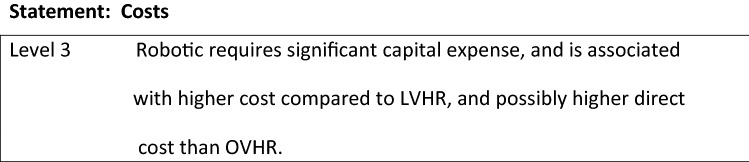



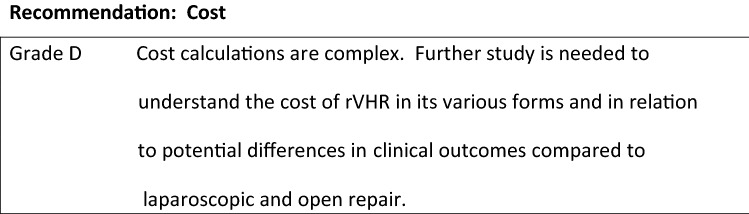


Cost is the factor most often used to critique robotic surgery across all disciplines. The capital cost of equipment alone often exceeds $2 million, with additional costs for service contracts, disposables, and instruments. In their review of the Vizient database, Armijo et al. found the cost of rVHR significantly greater than LVHR or OVHR [27]. Charges for RVHR were significantly higher as reported by the National Inpatient Sample as well [26]. However, charges and cost are different measures and cannot account for complexity of health care costs. Cost can vary greatly from hospital to hospital depending on payment contracts, Group Purchasing Organizations, type of procedure performed, coding, and reimbursement. Internationally, the diversity of health care organization is such that a single study will likely be unable to truly predict cost to any individual hospital or health system.

**Conflict of interest**

As with any new technology or technique, early literature must be interpreted in the context of the emerging technology itself, the larger changes in the given field of study, and the method of dissemination of the technique. Novel surgical techniques are quite often promulgated through industry, making author conflict of interest (COI) an important factor in interpreting the literature. In the case of rVHR, this is chiefly through Intuitive Surgical^®^ as this has been the only available robotic platform available for clinical use until recently. Patel et al. published an analysis of payments to published study authors from Intuitive Surgical^®^, finding that only 20.8% of authors disclosed payments from Intuitive, and nearly 64% of studies had at least one author who received payment despite no COI indicated. These discrepancies between reported and actual payments correlated with a higher likelihood of recommending robotic surgery [41]. This study was not specific to VHR, many of the articles cited in this work include statements of payment received from Intuitive Surgical^®^. While this does not automatically invalidate study findings, the discerning reader should carefully scrutinize study results. Author transparency is paramount.

Table 1A: Summary of Clinical Outcomes: IPOM and unspecified rVHR

**Table:** 

Clinical outcome	rIPOM	rVHR NOS
Total *n*	651 [1–3, 13, 15–18, 24, 37, 42]	1893 [12, 23, 25–27]
Operative time	74-180 min [1–3, 13, 16–18, 37, 42]	104.5 min [23]
Defect size	3–6.1 cm [1–3, 15–18, 37]	NR
Fascial closure	0–93% [1, 13, 15-18]	69.30% [12]
Length of stay	0.2–2.5 days [1–3, 13, 15–18, 24, 42]	1.1–4.3 days [12, 23, 25, 27]
SSO/minor complications	0–5% [1, 3, 15, 16, 18]	3.80% [12]
SSI/major complications	0–9% [1–3, 13, 15–18, 24, 37, 42]	0.8–1.7% [12, 26, 27]
Hernia recurrence	0–7.7% [2, 3, 13, 16, 17, 42]	NR

*SSO* surgical site occurrence, *SSI* surgical site infection, *IPOM* intraperitoneal onlay of mesh

Table 1B: Summary of clinical outcomes: rTAPP, rTAR/rRVHR, and eTEP

**Table:** 

Clinical Outcome	rTAPP	rTAR/rRVHR	eTEP
Total *n*	93 [36, 37, 43]	285 [14, 39, 40, 44, 45]	37 [46]
Operative time	73–163.7 min [36, 37, 43]	245–365 min [14, 40, 44, 45]	162 min [46]
Defect size	9.7–1219 cm^2^ [36, 37, 43]	6.5–13.5 cm [14, 39, 40, 44, 45]	7.4 cm [46]
Fascial closure	100% [36, 43]	96.3–100% [14, 39, 40, 44, 45]	100% [46]
Length of stay	0–1 [36, 43]	1–3.5 days [14, 39, 40, 44, 45]	0.7 days [46]
SSO/minor complications	0–3.7% [36, 37, 43]	0–52.8% [14, 39, 40, 44, 45]	5.40% [46]
SSI/major complications	0 [36, 43]	0–3.8% [14, 39, 40, 44, 45]	0 [46]
Hernia recurrence	0 [36, 43]	NR	NR

*SSO* surgical site occurrence, *SSI* surgical site infection, *rTAPP* robotic transabdominal preperitoneal repair, *rTAR* robotic transversus abdominis release, *rRVHR* Robotic retromuscular ventral hernia repair, *eTEP* extended totally extraperitoneal repair

**References** (in parentheses the level of evidence)

1. Ballantyne GH, Hourmont K, Wasielewski A (2003) Telerobotic laparoscopic repair of incisional ventral hernias using intraperitoneal prosthetic mesh. JSLS 7(1):7–14 (**4**)
2. Tayar C, Karoui M, Cherqui D, Fagniez PL (2007) Robot-assisted laparoscopic mesh repair of incisional hernias with exclusive intracorporeal suturing: a pilot study. Surg Endosc 21(10):1786–1789 (**4**)
3. Kudsi OY, Paluvoi N, Bhurtel P, McCabe Z, El-Jabri R (2015) Robotic Repair of Ventral Hernias: Preliminary Findings of a Case Series of 106 Consecutive Cases. Am J Robot Surg 2(1):22–26 (**4**)
4. Armijo PR, Pagkratis S, Boilesen E, Tanner T, Oleynikov D (2017) Growth in robotic-assisted procedures is from conversion of laparoscopic procedures and not from open surgeons’ conversion: a study of trends and costs. Surg Endosc 25:1127–1128 (**2c**)
5. Tandon A, Pathak S, Lyons NJR, Nunes QM, Daniels IR, Smart NJ (2016) Meta-analysis of closure of the fascial defect during laparoscopic incisional and ventral hernia repair. Br J Surg 103:1598–1607 (**2a**)
6. Clapp ML, Hicks SC, Awad SS, Liang MK (2013) Trans-cutaneous Closure of Central Defects (TCCD) in laparoscopic ventral hernia repairs (LVHR). World J Surg 37:42–51 (**3**)
7. Carter SA, Hicks SC, Brahmbhatt R, Liang MK (2014) Recurrence and pseudorecurrence after laparoscopic ventral hernia repair: predictors and patient-focused outcomes. Am Surg 80:138–148 (**3**)
8. Gonzalez AM, Romero RJ, Seetharamaiah R, Gallas M, Lamoureux J, Rabaza JR (2015) Laparoscopic ventral hernia repair with primary closure versus no primary closure of the defect: potential benefits of the robotic technology. Int J Med Robot 11:120–125 (**3**)
9. Chelala E, Baraké H, Estievenart J, Dessily M, Charara F, Allé JL (2015) Long-term outcomes of 1326 laparoscopic incisional and ventral hernia repair with the routine suturing concept: a single institution experience. Hernia 20(1):1–10 (**4**)
10. Banerjee A, Beck C, Narula VK, Linn J, Noria S, Zagol B, Mikami DJ (2012) Laparoscopic ventral hernia repair: does primary repair in addition to placement of mesh decrease recurrence? Surg Endosc 26:1264–1268 (**3**)
11. Orenstein SB, Dumeer JL, Monteagudo J, Poi MJ, Novitsky YW (2011) Outcomes of laparoscopic ventral hernia repair with routine defect closure using “shoelacing” technique. Surg Endosc 25:1452–1457 (**4**)
12. Gonzalez A, Escobar E, Romero R, Walker G, Mejias J, Gallas M, Dickens E, Johnson CJ, Rabaza J, Kudsi OY (2017) Robotic-assisted ventral hernia repair: a multicenter evaluation of clinical outcomes. Surg Endosc 31:1342–1349 (**4**)
13. Oviedo RJ, Robertson JC, Desai AS (2017) Robotic Ventral Hernia Repair and Endoscopic Component Separation: Outcomes. JSLS. 21(3):1–6 (**4**)
14. Warren JA, Cobb WS, Ewing JA, Carbonell AM (2017) Standard laparoscopic versus robotic retromuscular ventral hernia repair. Surg Endosc 31:324–332 (**3**)
15. Prabhu AS, Dickens EO, Copper CM, Mann JW, Yunis JP, Phillips S, Huang LC, Poulose BK, Rosen MJ (2017) Laparoscopic vs Robotic Intraperitoneal Mesh Repair for Incisional Hernia: An Americas Hernia Society Quality Collaborative Analysis. JACS 225(2):285–293 (**2c**)
16. Chen YJ, Huynh D, Nguyen S, Chin E, Divino C, Zhang L (2017) Outcomes of robot-assisted versus laparoscopic repair of small-sized ventral hernias. Surg Endosc 31:1275–1279 (**3**)
17. Walker PA, May AC, Mo J, Cherla DV, Santillan MR, Kim S, Ryan H, Shah SK,4, Wilson EB, Tsuda S (2018) Multicenter review of robotic versus laparoscopic ventral hernia repair: is there a role for robotics? Surg Endosc 93:1241–1245 (**3**)
18. Allison N, Tieu K, Snyder B, Pigazzi A, Wilson E (2012) Technical feasibility of robot-assisted ventral hernia repair. World J Surg 36:447–452 (**4**)
19. Liang MK, Clapp M, Li LT, Berger RL, Hicks SC, Awad S. (2013) Patient Satisfaction, chronic pain, and functional status following laparoscopic ventral hernia repair. World J Surg 37:530–537 (**4**)
20. Colavita PD, Tsirline VB, Belyansky I, Walters AL, Lincourt AE, Sing RF, Heniford BT (2012) Prospective, long-term comparison of quality of life in laparoscopic versus open ventral hernia repair. Ann Surg 256:714–722– discussion 722–3 (**2b**)
21. Muysoms F, Vander Mijnsbrugge G, Pletinckx P, Boldo E, Jacobs I, Michiels M, Ceulemans R (2013) Randomized clinical trial of mesh fixation with “double crown” versus “sutures and tackers” in laparoscopic ventral hernia repair. Hernia 17:603–612 (**1b**)
22. Bansal VK, Misra MC, Kumar S, Rao YK, Singhal P, Goswami A, Guleria S, Arora MK, Chabra A (2011) A prospective randomized study comparing suture mesh fixation versus tacker mesh fixation for laparoscopic repair of incisional and ventral hernias. Surg Endosc 25:1431–1438 (**1b**)
23. Oviedo RJ, Robertson JC, Alrajhi S (2016) First 101 Robotic General Surgery Cases in a Community Hospital. JSLS 20(3):1–6 (**4**)
24. Vasilescu D, Paun S (2012) Surgical treatment of parietal defects with “da Vinci” surgical robot. J Med Life 5:232–238 (**4**)
25. Altieri MS, Yang J, Xu J, Talamini M, Pryor A, Telem DA. (2018) Outcomes after Robotic Ventral Hernia Repair: A Study of 21,565 Patients in the State of New York. Am Surg 84:902–908 (**4**)
26. Coakley KM, Sims SM, Prasad T, Lincourt AE, Augenstein VA, Sing RF, Heniford BT, Colavita PD (2017) A nationwide evaluation of robotic ventral hernia surgery. Am J Surg 214:1158–1163 (**2c**)
27. Armijo P, Pratap A, Wang Y, Shostrom V, Oleynikov D (2017) Robotic ventral hernia repair is not superior to laparoscopic: a national database review. Surg Endosc 7:7–16. (**2c**)
28. Gray SH, Vick CC, Graham LA,, Finan KR, Neumayer LA, Hawn MT (2008) Risk of complications from enterotomy or unplanned bowel resection during elective hernia repair. Arch Surg 143:582–586 (**4**)
29. Halm JA, De Wall LL, Steyerberg EW, Jeekel J, Lange JF (2007) Intraperitoneal Polypropylene Mesh Hernia Repair Complicates Subsequent Abdominal Surgery. World J Surg 31:423–429 (**4**)
30. Patel PP, Love MW, Ewing JA, Warren JA, Cobb WS, Carbonell AM (2017) Risks of subsequent abdominal operations after laparoscopic ventral hernia repair. Surg Endosc 31:823–828 (**4**)
31. Jenkins ED, Yom V, Melman L, Brunt LM, Eagon JC, Frisella MM, Matthews BD (2010) Prospective evaluation of adhesion characteristics to intraperitoneal mesh and adhesiolysis-related complications during laparoscopic re-exploration after prior ventral hernia repair. Surg Endosc 24:3002–3007 (**3**)
32. Snyder CW, Graham LA, Gray SH, Vick CC, Hawn MT. (2011) Effect of mesh type and position on subsequent abdominal operations after incisional hernia repair. JACS 212:496–502. discussion 502–504 (**4**)
33. Steinhagen E, Khaitov S, Steinhagen RM (2010) Intraluminal migration of mesh following incisional hernia repair. Hernia 14:659–662. Level 4 evidence
34. Prasad P, Tantia O, Patle NM, Khanna S, Sen B. (2011) Laparoscopic transabdominal preperitoneal repair of ventral hernia: a step towards physiological repair. Indian J Surg 73:403–408 (**4**)
35. Hilling DE, Koppert LB, Keijzer R, Stassen LP, Oei IH (2009) Laparoscopic correction of umbilical hernias using a transabdominal preperitoneal approach: results of a pilot study. Surg Endosc 23:1740–1744 (**4**)
36. Orthopoulos G, Kudsi OY (2018) Feasibility of Robotic-Assisted Transabdominal Preperitoneal Ventral Hernia Repair. J Laparoendosc Adv Surg Tech A 28:434–438 (**4**)
37. Kennedy M, Barrera K, Akelik A, Constable Y, Smith M, Chung P, Sugiyama G (2018) Robotic TAPP Ventral Hernia Repair: Early Lessons Learned at an Inner City Safety Net Hospital. JSLS 22(1):1–5 (**3**)
38. Abdalla RZ, Garcia RB, Costa R, Luca CR, Abdalla BM. (2012) Procedimento de Rives/Stoppa modificado robô-assistido para correção de hérnias ventrais da linha média. Arq Bras Cir Dig 25(2):129–132 (**4**)
39. Carbonell AM, Warren JA, Prabhu AS, Ballecer CD, Janczyk RJ, Herrera J, Huang LC, Phillips S, Rosen MJ, Poulose BK (2018) Reducing Length of Stay Using a Robotic-assisted Approach for Retromuscular Ventral Hernia Repair: A Comparative Analysis From the Americas Hernia Society Quality Collaborative. Ann Surg 267:210–217 (**2c**)
40. Bittner JG, Alrefai S, Vy M, Mabe M, Del Prado PAR, Clingempeel NL (2017) Comparative analysis of open and robotic transversus abdominis release for ventral hernia repair. Surg Endosc 16:179–188 (**3**)
41. Patel SV, Yu D, Elsolh B, Goldacre BM, Nash GM (2018) Assessment of conflicts of interest in robotic surgical studies: validating author’s declarations with the open payments database. Ann Surg 268:86–92 (**2B**)
42. Gonzalez AM, Romero RJ, Seetharamaiah R, Gallas M, Lamoureux J, Rabaza JR (2015) Laparoscopic ventral hernia repair with primary closure versus no primary closure of the defect: potential benefits of the robotic technology. Int J Med Robot 11:120–125 (**3**)
43. Sugiyama G, Chivukula S, Chung PJ, Alfonso A (2015) Robot-Assisted Transabdominal Preperitoneal Ventral Hernia Repair. JSLS. 19(4):1–3 (**4**)
44. Martin-del-Campo LA, Weltz AS, Belyansky I, Novitsky YW3. (2017) Comparative analysis of perioperative outcomes of robotic versus open transversus abdominis release. Surg Endosc 204:709–716 (**3**)
45. Halka JT, Vasyluk A, DeMare AM, Janczyk RJ, Iacco AA (2018) Robotic and hybrid robotic transversus abdominis release may be performed with low length of stay and wound morbidity. Am J Surg 215:462–465 (**3**)
46. Belyansky I, Reza Zahiri H, Sanford Z, Weltz AS, Park A (2018) Early operative outcomes of endoscopic (eTEP access) robotic-assisted retromuscular abdominal wall hernia repair. Hernia 22(5):837–847. 10.1007/s10029-018-1795-z. Epub 2018 Jul 4 (**4**)

## Chapter 7. Key question: treatment of lateral primary or incisional hernias: Which technique should be preferred?

### W. Reinpold, B. Sutedja

**Search terms**

Lateral abdominal wall hernia, lateral eventration, iliac hernia, subcostal hernia, flank hernia repair, flank hernia repair with mesh, lumbar hernia repair, lumbar hernia repair with mesh, unusual hernias of the abdominal wall, spigelian hernia, spigelian hernia repair, lateral incisional hernia, traumatic lumbar hernia, Grynfelt OR Grynfelt’s hernia, Petit OR Petit’s hernia; the above AND laparoscopy, lumbar hernia AND lumbar muscles AND paralysis, lumbar hernia AND lumbar muscles AND paralysis AND bulge, lumbar hernia AND lumbar muscles AND paralysis AND nephrectomy, lumbar hernia AND nephrectomy.

**Searching machines**

PubMed, Embase, and Medline

(2000–2018) were searched. For the study of the old guidelines read the original publication in “Surg Endosc (2014) 28: page 399-401”.


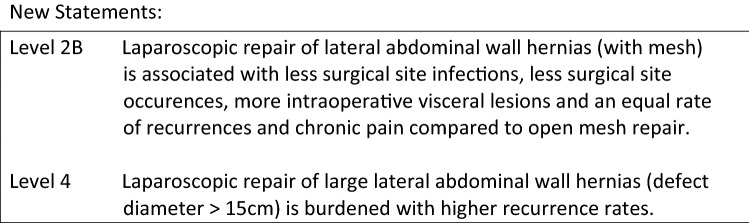



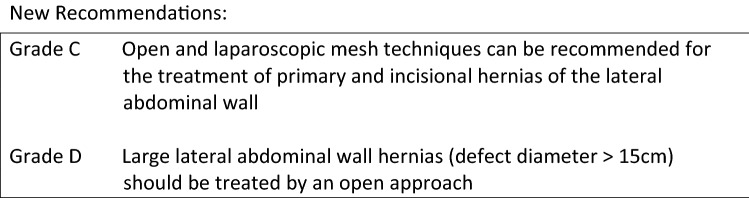


**Introduction**

Lateral abdominal wall hernias comprise primary and secondary defects of the lumbar, subcostal, flank, and iliac region (EHS classification). Compared to midline hernias (hernias of the rectus compartment) primary and incisional lateral abdominal wall hernias are rare. Consequently, the evidence guiding the surgical treatment of lateral abdominal wall hernias is scant. The lumbar region is divided into the superior and inferior lumbar space. Primary lumbar hernias of the superior space are denominated Grynfeltt hernia and those of the lower space Petit hernia. The boundaries of the inferior lumbar hernia are the latissimus dorsi muscle posteriorly, the external oblique muscle anteriorly, and the iliac crest inferiorly. The boundaries of the superior lumbar hernia are the 12th rib superiorly, the internal oblique muscle anteriorly, and the erector spinae muscle posteriorly.

Lateral hernias often are located in more than one region of the lateral compartment.

The first IEHS guidelines included 12 papers, 11 case series with more than five patients and one small RCT which included 16 patients and compared open with laparoscopic repair. In total 123 patients were evaluated.

The new literature search (2012–2018) revealed 10 case series (8 retrospective) which included 439 patients on open operations of lateral abdominal wall hernias [1–10] and 4 retrospective case series with 188 patients on laparoscopic repairs ([11–14]; Table 1 + 2).

The level of evidence of all trials was 4 and there were no reports on suture repair. The infection rate after open repair varied between 0 and 25% while no infections were reported after laparoscopic operations. The rate of intraoperative visceral damage was 0 to 4% in open and 0 to 15% in laparoscopic repair, respectively. One retrospective case series of 73 patients with laparoscopic repair of lateral incisional hernias with a medium follow-up of 62 months reported a total recurrence rate of 8% and 25% in the subgroup of subcostal incisional hernias [11]. The authors concluded that a defect size of > 15 cm was a risk factor of recurrence. Only four case series reported on chronic pain [5, 7, 8, 10]. However, according to a systematic review [15] chronic pain rates after open and laparoscopic lateral abdominal wall hernia repair seem to be comparable.

Table 1: Publications on open lateral abdominal wall hernia repair 2012 to 2018 [1–10]

**Table:** 

Author	Type of trial	Type of hernia	Type of repair	Number (n)	Complications (%)	Recurrence (%)	Chronic pain (%)	Mean Follow-up months	Miscell.
Moreno-Egea (2015)	Retrospective case series	Complex lateral hernias	Double prosthetic repair	53	25	0	NR	NR	Mean defect diameter 18 cm
Phillips (2012)	Retrospective case series	Flank hernias	Retromuscular	16	25 (Infections *n* = 3, Ureter lesion *n* = 1)	0	NR	17	9 incarcerated hernias
Veyrie (2013)	Retrospective case series	Lateral incisional hernias	Retromuscular with poyester mesh	61 Subcostal 14 Flank 12 Iliac 35	18 (*n* = 4 reoperations)	6.6	NR	47	Mean defect size 56 cm^2^
Luc (2014)	Prospective comparative trial	Lateral incisional hernias	Open retromuscular or IPOM	112 61 after renal transplantation	24 (24.5 after renal transplant. versus 23.5)	10 versus 10	NR		Incisional hernias after renal Tx or no renal Tx
Peres (2014)	Retrospective case series	Subcos-tal incisional hernias	open	25	33 (*n* = 8)	4	4		
Blair (2015)	Prospective case series	Lateral incisional hernias	Sublay/underlay with acell dermal matrix	20 (lumbar 10, suprapub. 7, iliac 3)	15	0	NR	24	Mean defect size 270 cm2
Pezeshk (2015)	Retrospective case series	Lateral incisional hernias	Sublay/underlay with acell dermal matrix	29	31	3	10	21	
Purnell (2016)	Retrospective case series	Flank hernias	Open IPOM = 19 Open Interpar-Ietal = 12	31	0 infections	3	3	27	
Patel (2016)	Retrospective case series	Lateral Incisional hernias	Open retromus 50%, preperit 41%, IPOM 7% Onlay 2%	61 Subcostal 14 Flank 33 iliac 11 Lumbar 3	SSO 49% SSI 13%	12	NR	15	Mean defect size 79 cm2
Renard (2017)	Retrospective case series	Lumbar Incisional hernia	Large Retromus Mesh with large overlap	31	32	7	10		Post Nephr- ectomy N = 20
Total articles (N = 10)				439					

Table 2: Publications on laparoscopic lateral abdominal wall hernia repair 2012 to 2018 [11–14]

**Table:** 

Author	Type of trial	Type of hernia	Type of repair	Number (*n*)	Complications (%)	Recurrence (%)	Chronic pain (%)	Mean Follow-up months	Miscell.
Moreno-Egea (2012)	Retrospective case series	Lateral Incisional Hernias (subcostal, Iliac, lumbar)	Laparo- sopic IPOM	73		8 subcostal 25		62	Predictor for recurrence defect diameter >15 cm
Lal (2014)	Retrospective case series	Lateral Incisional hernias	Laparo- Sopic IPOM	25 lumbar 5, suprapub 7, iliac 10, subsostal 3	Intraoperative lesions 15%, total 25%	4 (n = 1, iliac)			
Farrarese (2016)	Retrospective case series	Lateral Incisional Hernias (subcostal, flank, Iliac, lumbar)	Laparo- sopic IPOM	76					
Novitsky (2017)	Retrospective case series	Traumatic Flank hernias	Laparo- sopic IPOM	14	0	0		35	N = 11 chronic incarcerated
Total articles N = 4				188					

**Spieghelian hernias**


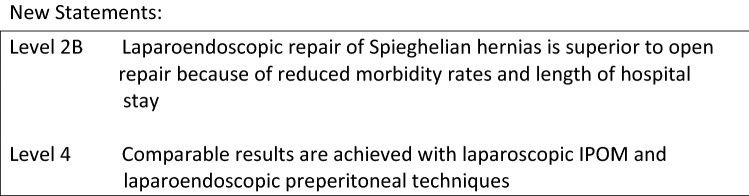



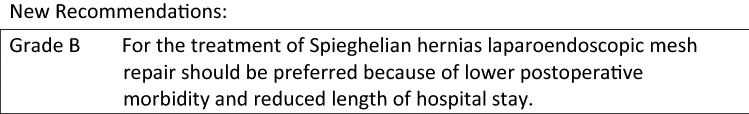


The Spiegelian hernia first escribed anatomically by Adriaan van den Spieghel (1578–1625) is located at the level of the semicircular line where the fascias of the oblique and transversus muscles begin to split into separate layers of the abdominal musculature. Spiegelian hernias (SH) account for 1% to 2% of abdominal wall hernias. Since the advent of minimally invasive surgery laparoscopic methods have become increasingly popular with various techniques being described in the literature. Since 2012 thirtytwo case series with 5 or more patients were included in this review. No randomized-controlled trials on the treatment of SH were identified. One systematic review was published in 2016 [4] which included 237 SHs that were repaired by various techniques. Intraperitoneal onlay mesh technique was the most popular repair method with minimal complications and recurrences reported in all techniques.

**Conclusions**

There are a number of laparoscopic techniques available to the surgeon repairing a SH. Overall, laparoscopic repair of the SH is a safe and acceptable method.

**References** (in parentheses the level of evidence)

1. Moreno-Egea A (2015) Double Prosthetic Repair for Complex Incisional Hernia Repair: Long-term Results and Evolution of the Technique. Am Surg 81(11):1138–1143. (**4**)
2. Phillips MS, Krpata DM, Blatnik JA, Rosen MJ. (2012) Retromuscular preperitoneal repair of flank hernias J Gastrointest Surg. 16(8):1548–1553. (**4**)
3. Veyrie N, Poghosyan T, Corigliano N, Canard G, Servajean S, Bouillot JL. (2013) Lateral incisional hernia repair by the retromuscular approach with polyester standard mesh: topographic considerations and long-term follow-up of 61 consecutive patients. World J Surg 37(3):538–544. (**4**)
4. Luc G, David A, Couzi L, Midy D, Collet D, Dubuisson V. (2014) Lateral incisional hernia after renal transplantation: a comparative study. World J Surg 38(11):2791–2796. (**4**)
5. Peres MA, Aguiar HR, Andreollo NA (2014) Surgical treatment of subcostal incisional hernia with polypropylene mesh-analysis of late results. [Article in English, Portuguese] Rev Col Bras Cir. 41(2):82–86. (**4**)
6. Blair LJ, Cox TC, Huntington CR, Ross SW, Kneisl JS, Augenstein VA, Heniford BT. (2015) Bone Anchor Fixation in Abdominal Wall Reconstruction: A Useful Adjunct in Suprapubic and Para-iliac Hernia Repair. Am Surg 81(7):693–697. (**4**)
7. Pezeshk RA, Pulikkottil BJ, Bailey SH, Schaffer NE, Reece EM, Thornton NJ, Gupta AR, Hoxworth RE. (2015) An Evidence-Based Model for the Successful Treatment of Flank and Lateral Abdominal Wall Hernias. Plast Reconstr Surg 36(2):377–385. (**4**)
8. Purnell CA, Park E, Turin SY, Dumanian GA. (2016) Postoperative Flank Defects, Hernias, and Bulges: A Reliable Method for Repair. Plast Reconstr Surg 137(3):994–1001 (**4**)
9. Patel P, Warren JA, Mansour R, Cobb WS 4^th^ (2016) A Large Single-Center Experience of Open Lateral Abdominal Wall Hernia Repairs. Am Surg 82(7):608–612. (**4**)
10. Renard Y, de Mestier L, Cagniet A, Demichel N, Marchand C, Meffert JL, Kianmanesh R, Palot JP (2017) Open retromuscular large mesh reconstruction of lumbar incisional hernias including the atrophic muscular area. Hernia 21(3):341–349. (**4**)
11. Moreno-Egea A, Carrillo-Alcaraz A. (2012) Management of non-midline incisional hernia by the laparoscopic approach: results of a long-term follow-up prospective study. Surg Endosc 26(4):1069–78. (**4**)
12. Lal R, Sharma D, Hazrah P, Kumar P, Borgharia S, Agarwal A. (2014) Laparoscopic management of nonmidline ventral hernia. J Laparoendosc Adv Surg Tech A 24(7):445–450. (**4**)
13. Farrarese A, Enrico S, Solej M, Surace A, Nardi MJ, Millo P, Allieta R, Feleppa C, D’Ambra L, Berti S, Gelarda E, Borghi F, Pozzo G, Marino B, Marchigiano E, Cumbo P, Bellomo MP, Filippa C, Depaolis P, Nano M, Martino V (2016) Laparoscopic management of non-midline incisional hernia: A multicentric study. Int J Surg 33 Suppl 1:S108–S113. (**4**)
14. Novitsky YW (2018) Laparoscopic repair of traumatic flank hernias. Hernia 22(2):363–369. 10.1007/s10029-017-1707-7. Epub 2017 Dec 15. (**4**)
15. Zhou DJ, Carlson MA (2018) Incidence, etiology, management, and outcomes of flank hernia: review of published data. Hernia 22(2):353–361. 10.1007/s10029-018-1740-1. Epub 2018 Jan 27 (**2B**)
16. Barnes TG, McWhinnie DL (2016) Laparoscopic Spigelian Hernia Repair: A Systematic Review. Surg Laparosc Endosc Percutan Tech. 26(4):265-70. (**2B**)

## Chapter 8. Education and training in laparoscopic ventral hernia repair

### D. Lomanto, Hrishikesh P. Salgaonkar

**Search terms**

Hernia/abdominal surgery/Ventral hernia, Umbilical, Incisional hernia, learning curve, Education/Laparoscopy, General surgery/education, Surgical procedures/operative education, Surgical procedures/operative psychology, Teaching/methods, Internship/residency, Competency-based education, Computer assisted instruction.

**Searching machines**

PubMed, Embase, Medline and Cochrane Library (2003–2017) were searched for studies for potential inclusion. For the study of the original guidelines read the publication in “Surg Endosc (2014) 28:401–403”.

**New publications**

In addition to studies included in the published guidelines, total of 5 new studies were found and included in the update. One Level 1 study, one level 2 study, and three Level 3 studies are supplementing the knowledge of Education and training in laparoscopic ventral or incisional hernia repair.

New Statement: identical to previous except statement below:
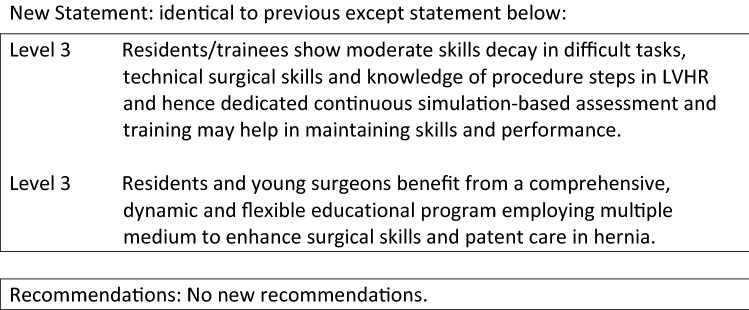


Comments: We identified one new study defining learning curve in Laparoscopic ventral hernia repair (LVHR) [1]. In this study, results of LVHR performed by three experienced surgeons in a single center were retrospectively analyzed. They found that after 20 cases the overall performance plateaued, most notably in intra and postoperative complications. The operative time stabilized after 12 cases.

The SAGES Hernia task force conducted a study using interviews and online surveys amongst the task force members, chief residents, fellows, and surgical residents [2]. They commented that the traditional “see one, do one, teach one” method and prevalent methods of training is inadequate for learning hernia repair. In addition to supervised surgery, most trainees prefer new learning methods such as simulation, web-based training, hands on laboratory, and master videos. The consensus was that educational programs should be comprehensive, dynamic, and flexible to employ various media to address the deficits in hernia surgery training and patient care.

D’Angelo et al., in their multi-center study, tried to evaluate effect of time away from clinical work on clinical skills during dedicated research rotations in surgical residency [3]. Simulation-based training in LVHR, along with others procedures was used to assess improvements in perception of skill decay. They concluded that most residents during their research postings expect moderate skills decay in LVHR, and hence suggested to incorporate simulation-based training in the curriculum during dedicated research time or research fellowships to maintain trainees' surgical skills. Sonnadara et al. similarly suggested that competency-based training rather than time spent in training should be used to assess a surgeon’s skill level [4]. A Cochrane systemic review suggested that virtual reality training when compared to no training or box-trainer training reduces operating time and improves operative performance of surgical trainees with limited laparoscopic experience [5].

In today’s day and age, it is necessary for residency and fellowship training programs to incorporate simulation-based training and virtual reality training in the curriculum along with surgical training under supervision. Specific simulation training for ventral and incisional hernia repair will benefit the trainee to gain hernia specific skills and also prevent skill decay.

**References** (in parentheses graduation of evidence)

1. Al-Harazi A, Goel R, Tan CT, Cheah WK, Lomanto D (2014) Laparoscopic ventral hernia repair: defining the learning curve. Surg Laparosc Endosc Percutan Tech 24(6):475–477. PMID: 24743667 (**3**)
2. Zahiri HR, Park AE, Pugh CM, Vassiliou M, Voeller G (2015) “See one, do one, teach one”: inadequacies of current methods to train surgeons in hernia repair. Surg Endosc 29(10):2867–2872. Epub 2015 Jul 22. PMID: 26198155. (**3**)
3. D’Angelo A-LD, Ray RD, Jenewein CG, Jones GF, Pugh CM (2015) Residents’ Perception of Skill Decay during Dedicated Research Time. J Surg Res 199(1):23–31. Epub 2015 Jun 23. PMID: 26197949 (**2b**)
4. Sonnadara RR, Mui C, McQueen S, Mironova P, Nousiainen M, Safir O, Kraemer W, Ferguson P, Alman B, Reznick R (2014) Reflections on competency-based education and training for surgical residents. J Surg Educ 71(1):151–158. Epub 2013 Sep 14. PMID: 24411437. (**3**)
5. Nagendran M, Gurusamy KS, Aggarwal R, Loizidou M, Davidson BR (2013) Virtual reality training for surgical trainees in laparoscopic surgery. Cochrane Database of Systematic Reviews Issue 8. Art. No.: CD006575. 10.1002/14651858.cd006575.pub3. (**1a**)

